# Crystal Structure of the Gamma-2 Herpesvirus LANA DNA Binding Domain Identifies Charged Surface Residues Which Impact Viral Latency

**DOI:** 10.1371/journal.ppat.1003673

**Published:** 2013-10-17

**Authors:** Bruno Correia, Sofia A. Cerqueira, Chantal Beauchemin, Marta Pires de Miranda, Shijun Li, Rajesh Ponnusamy, Lénia Rodrigues, Thomas R. Schneider, Maria A. Carrondo, Kenneth M. Kaye, J. Pedro Simas, Colin E. McVey

**Affiliations:** 1 Instituto de Tecnologia Química e Biológica, Universidade Nova de Lisboa, Oeiras, Portugal; 2 Instituto de Microbiologia e Instituto de Medicina Molecular, Faculdade de Medicina, Universidade de Lisboa, Lisboa, Portugal; 3 Departments of Medicine, Brigham and Women's Hospital and Harvard Medical School, Boston, Massachusetts, United States of America; 4 EMBL c/o DESY, Hamburg, Germany; University of Southern California Keck School of Medicine, United States of America

## Abstract

Latency-associated nuclear antigen (LANA) mediates γ2-herpesvirus genome persistence and regulates transcription. We describe the crystal structure of the murine gammaherpesvirus-68 LANA C-terminal domain at 2.2 Å resolution. The structure reveals an alpha-beta fold that assembles as a dimer, reminiscent of Epstein-Barr virus EBNA1. A predicted DNA binding surface is present and opposite this interface is a positive electrostatic patch. Targeted DNA recognition substitutions eliminated DNA binding, while certain charged patch mutations reduced bromodomain protein, BRD4, binding. Virus containing LANA abolished for DNA binding was incapable of viable latent infection in mice. Virus with mutations at the charged patch periphery exhibited substantial deficiency in expansion of latent infection, while central region substitutions had little effect. This deficiency was independent of BRD4. These results elucidate the LANA DNA binding domain structure and reveal a unique charged region that exerts a critical role in viral latent infection, likely acting through a host cell protein(s).

## Author Summary

Herpesviruses establish life-long latent infections. During latency, gammaherpesviruses, such as Kaposi's sarcoma-associated herpesvirus (KSHV), persist as multicopy, circularized genomes in the cell nucleus and express a small subset of viral genes. KSHV latency-associated nuclear antigen (LANA) is the predominant gene expressed during latent infection. C-terminal LANA binds KSHV terminal repeat (TR) DNA to mediate DNA replication. TR DNA binding also allows tethering of the viral genome to mitotic chromosomes to mediate DNA segregation to daughter nuclei. We describe here the crystal structure of the murine gammaherpesvirus 68 LANA DNA binding domain, which is homologous to that of KSHV LANA. The structure revealed a dimer and we identified residues involved in the interaction with viral DNA. Mutation of these residues abolished DNA binding and viable latency establishment in a mouse model of infection. We also identified a positively charged patch on the dimer surface opposite to the DNA binding region and found this patch exerts an important role in the virus's ability to expand latent infection in vivo. This work elucidates the structure of the LANA DNA binding domain and identifies a novel surface feature that is critical for viral latent infection, likely by acting through a host cell protein.

## Introduction

Herpesviruses are ubiquitous viruses, which infect many mammalian species and are a leading cause of human viral disease. There are two known human viruses from the gamma herpesvirus sub-family. The Epstein-Barr virus (EBV), a gamma-1 herpesvirus (Lymphocryptovirus), and the Kaposi's sarcoma-associated herpesvirus (KSHV), a gamma-2 herpesvirus (Rhadinovirus). KSHV has an etiologic role in Kaposi's Sarcoma, the leading AIDS malignancy, as well as primary effusion lymphoma and multicentric Castleman's disease. EBV is also associated with human cancers. These human viruses do not readily infect small laboratory animals. The murine gammaherpesvirus 68 (MHV-68 or murid herpesvirus 4), a rhadinovirus that was isolated from naturally infected rodents, is structurally and functionally related to human gamma-herpesviruses and readily infects mice, thus providing a mouse model for the investigation of gammaherpesvirus pathogenesis [1]–[3].

A key feature of herpesvirus infection is their lifelong persistence in the host in the form of latency. During latent infection, viral genomes persist as multi-copy, circularized, extrachromosomal episomes (plasmids). Only a small subset of viral genes is expressed during latency. In the case of gamma-herpesvirus sub-family members, latent infection is predominantly established in B lymphocytes. Intransal infection of mice with MHV-68 causes an acute self-limiting respiratory infection, followed by the establishment of splenic latency, which peaks 14 days after infection [4], [5]. Splenic germinal centre (GC) B cells have a key role both in the establishment and maintenance of viral latency [6]. Like EBV [7], MHV-68 establishes latency predominantly in activated germinal centre B cells. This strategy favours physiological access of the virus to the memory B-cell pool, the major reservoir of long-term latency.

KSHV latency-associated nuclear antigen (kLANA), a 1,162 amino acid protein encoded by *ORF73* of the viral genome, is the predominant gene expressed in latent infection. LANA mediates persistence of episomal DNA and thus is required for long-term maintenance of viral genomes in dividing cells, a role central to viral latency. LANA promotes episomal replication and segregation to progeny nuclei after mitosis. The C-terminal region of kLANA harbours a DNA-binding domain (DBD) that self-associates and binds to terminal repeat (TR) DNA sequences of the viral episome [8]–[14]. LANA cooperatively binds to two sites within the TR DNA, LBS1 and LBS2. LBS1 is a high affinity site capable of facilitating the cooperative binding of LANA to LBS2. Both sites contribute to the ability of LANA to suppress transcription and to facilitate DNA replication [10]. An N-terminal region of kLANA is able to attach to chromosomes through binding to histones H2A and H2B [15]. Together, these binding properties allow kLANA to tether the viral genome to chromosomes during mitosis thus ensuring segregation of the episome to daughter nuclei [16], [17]. Association of viral genomes with host mitotic chromosomes via a virus-encoded protein is a strategy employed by a number of different latent DNA viruses. Similar to the KSHV LANA, EBV EBNA1 and papillomavirus E2 proteins play a role in viral genome maintenance [18]–[22].

All gamma-2 herpesviruses encode homologs of KSHV LANA. The *ORF73* from MHV-68 encodes a much smaller, 314 amino acid, 50 kDa nuclear protein (termed mLANA thereafter) which lacks the extensive internal acidic and glutamine-rich repeat region of kLANA. The C-terminal region of mLANA, comprising amino acid residues 140 to 263, has amino acid similarity to the kLANA DNA-binding domain. Similar to kLANA, mLANA was recently shown to act on TR elements of the MHV-68 genome to mediate episome maintenance and to associate with mitotic chromosomes [23], and mLANA DNA-binding sites have been identified within MHV-68 TR DNA [24]. In a mouse model of infection, latency-associated nuclear antigen (LANA) protein is selectively expressed in infected GC B cells [25]. Recombinant viruses that do not express mLANA [26], [27], or bear disruptive mutations in the predicted DNA-binding domain fail to establish viable latency [24]. mLANA therefore performs functions equivalent to those of kLANA regarding episomal maintenance and is essential for establishment of viable latency *in vivo*.

LANA proteins are also modulators of transcription from a variety of cellular and viral promoters and affect cellular growth [28], [29]. Both kLANA and mLANA are able to regulate transcription through E3-ubiquitin ligase activity [30]–[32]. mLANA promotes the polyubiquitination and subsequent proteasomal-dependent nuclear degradation of host nuclear factor-kappa B (NF-κB) [31]. The mechanism involves the assembly of an Elongin C/Cullin5/SOCS (suppressors of cytokine signalling)-like complex, mediated by an unconventional viral SOCS-box motif (residues 199–206), homologous to the kLANA Cul5 box, present in the C-terminal domain of mLANA [30], [31]. Another motif also within the C-terminal region of mLANA (QAKKLK motif, residues 226–231) has been shown to bind to several members of the BET (Bromodomain and Extra Terminal domain) family of proteins, including BRD2, BRD3 and BRD4 [33], [34], that interact with acetylated histones. mLANA interaction with BET proteins leads to activation of cell cycle promoters.

Secondary structure predictions have suggested that the C-terminal regions of both mLANA and kLANA have similarity to the X-ray structure of the DNA-binding domain of the EBV nuclear antigen 1 protein (EBNA1) [35]. As a first step towards understanding the mechanism by which the C-terminal region of mLANA exerts its functions, we have determined the crystal structure of this mLANA domain (amino acids 140–272). mLANA_140–272_ forms a β-barrel induced dimer and has an overall fold with an α-helix arrangement similar to that of the EBNA1 and E2 latency protein structures [36], [37]. However, the structure reveals unique features that exert important effects on viral persistence in an animal model.

## Results

### X-ray crystal structure of C-terminal mLANA reveals an alpha+beta fold that assembles to form a dimer

**Figure 1:**
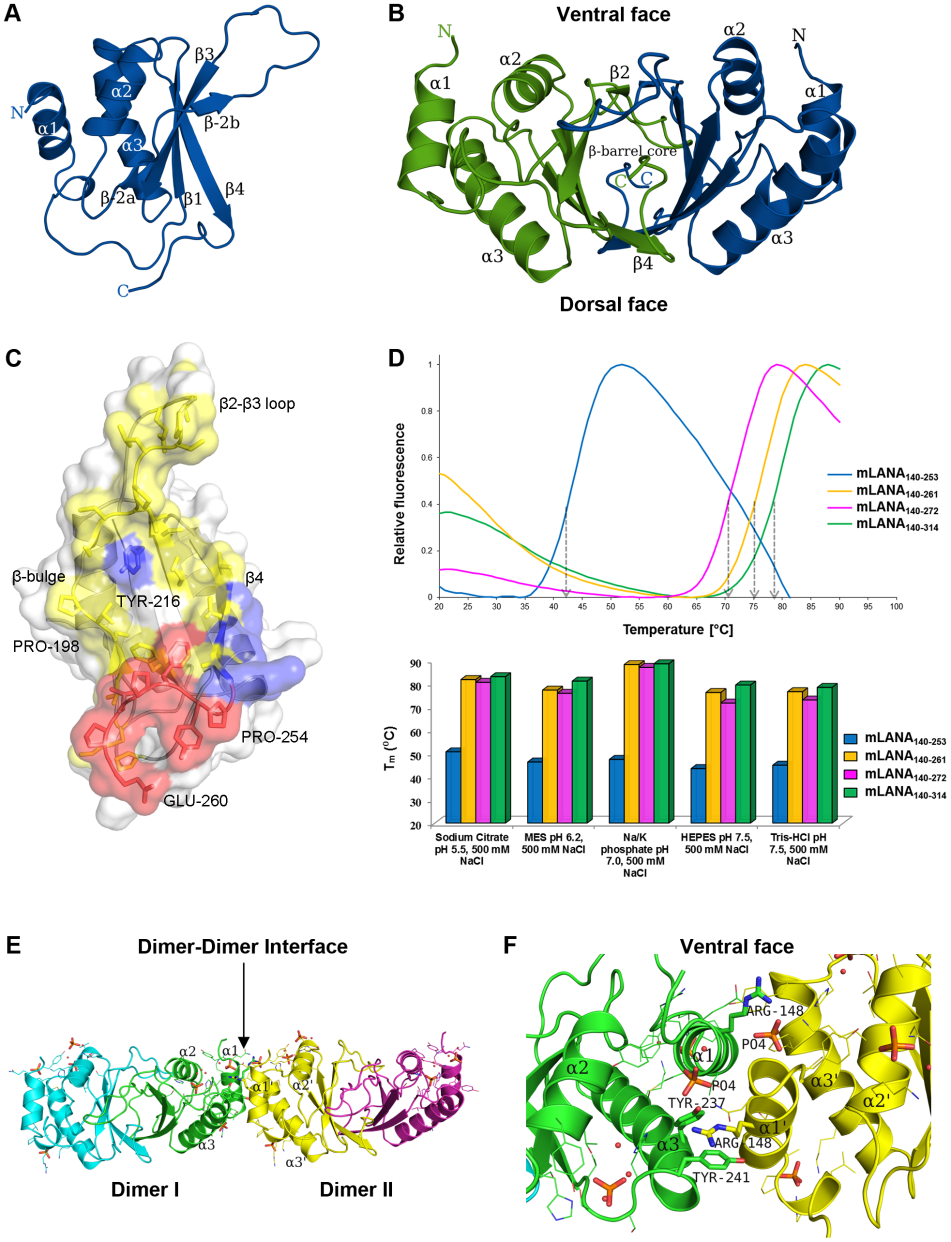
Structure of the C-terminal domain of mLANA. (**A**) A ribbon representation with secondary structure assignment for a monomer of the MHV-68 mLANA_140–272_ DNA-binding domain (see also Figure S1). (**B**) A representation of the mLANA_140–272_ quaternary structure and its dimeric conformation viewed along the non-crystallographic axis and illustrates the α-helical arrangement around the β-barrel core. The two chains are coloured in blue (chain A) and green (chain B). (**C**) Cross section of the dimer interface with coloured surface properties. A large area (∼1440 Å^2^) constituting the hydrophobic core is displayed in yellow with the hydrogen bonding residues displayed in blue. Deleting residues P254–P259 in red from the C-terminus destabilizes the dimer, as it contributes to the hydrophobic stability of the dimer interface (see also Figure S2). (**D**) Fluorescence-based thermal shift analysis of mLANA truncations (arrows display the midpoint of the unfolding transitions). The midpoint melting temperatures of the protein-unfolding transition (*T_m_*) for mLANA DBD truncations are shown for the optimal buffers. (**E**) mLANA tetramer assembly presumed for cooperative TR DNA binding as observed in the crystal packing with the symmetry equivalent dimer. Phosphate interactions coordinate the dimer-dimer interface. Two dimers form a tetramer stabilized by an interface with a surface area (∼535 Å^2^) per monomer that is approximately 12% of that of the total surface of a single subunit (∼7,500 Å^2^). (**F**) A ribbon representation of the dimer-dimer interface view from the ventral side. In the interface Arg-148 from dimer-2 stacks against Tyr-241, and makes a weak hydrogen bond with Tyr-237 from dimer-1. The arginine residue is conserved in both mLANA and kLANA proteins which has the ability to intercalate between hydrophobic aromatic amino acids on the second dimer.

The structure of the C-terminal domain of mLANA was determined at 2.2 Å resolution. Data collection and refinement statistics are reported in Table 1. The initial structure was solved using the coordinates of the EBV EBNA1 dimer, which has both functional and predicted secondary structure homology, as a molecular replacement model [36]. The crystallized domain contained residues 140–272, designated mLANA_140–272_ (Figure S1). The refined electron density map was readily interpretable with exceptions only at the termini and loop regions. The tertiary structure of mLANA_140–272_ exhibits an α+β ferredoxin-like fold (Figure 1A) which assembles to form a dimeric eight-stranded anti-parallel β-barrel that is central to its functional architecture. This dimer can be viewed from opposite sides of the β-barrel core which for clarity is termed the ventral (top) and the dorsal sides (bottom) (Figure 1B). Each monomer contributes an anti-parallel 4-stranded β-sheet “half-barrel” from which helix α2 and α3 pack onto the plaited sheet with their axis parallel to the strands of the sheet. The core of the barrel is occupied by large hydrophobic surface (Figure S2) that contributes to the stability of mLANA (Figure 1C). Size exclusion analysis confirms that mLANA_140–272_ is also a dimer in solution (Figure S2B and C). When the dimer interface was disrupted by removal of residues 254–261, which lie below the β2–β3 loop of the adjacent monomer, the melting temperature of mLANA_140–253_ decreased drastically (T_m_ 43°C), indicating the importance of these residues for stability (Figure 1D). The flanking helix α1 caps this helical arrangement and lies perpendicular to the central β-barrel. Helix α2, situated on the ventral side, is postulated to be the DNA recognition helix (see below).

**Table 1:** Data collection and refinement statistics.

**X-ray diffraction data**	
Wavelength (Å)	1.129
Space group	C2
Cell dimensions: a, b, c (Å)	88.6 61.7 63.7
α, β, γ (°)	90.0, 99.4, 90.0
Resolution (Å)	37.6–2.2 (2.32–2.20)
R_merge_	0.059 (0.64)
R_meas_/R_pim_	0.063 (0.69)/0.023 (0.25)
*I/σI*	21.4 (3.3)
Completeness (%)	99.9 (98.5)
Redundancy	7.5 (7.4)
Total measured reflections	130,451
Unique reflections	17,333 (2,507)
Wilson B-factor (Å^2^)	42.3
**Refinement**	
Resolution (Å)	30.8–2.2
*R_work_/R_free_*	0.1714/0.2032
Atoms: protein (chain A)/protein (chain B)/PO4^2−^/water	1979/1946/35/62
B factors: chain A/chain B/PO4/water	49.4/50.7/51.8/41.9
Rmsd bond length (Å)	0.007
Rmsd bond angles (°)	1.168
Ramachandran analysis: favoured/allowed (%)	93.0/7.0

*R*_work_ and *R*_free_ are defined by *R* = ∑*hkl*∥*F_obs_*|−|*F*_calc_∥/∑*hkl*|*F*_obs_|, where *hkl* are the indices of the reflections (used in refinement for *R*_work_; 5%, not used in refinement, for *R*_free_), and *F*_obs_ and *F*_calc_ are the structure factors deduced from measured intensities and calculated from the model, respectively. Values in parentheses are for the highest resolution shell.

LANA cooperatively binds to two adjacent sites within TR DNA, similar to the EBV DS element required for EBNA1 binding [38], suggesting a dimer-dimer interaction [39]. Analysis of the mLANA crystal packing shows that dimers pack so that the DNA-binding flanking helix α1 in each dimer is in position to interact with both the flanking helix α1 and helix α3 from the adjacent dimer (Figure 1E). Two phosphate ions on the ventral surface contribute and coordinate the binding of the α1–α3′ dimer-dimer interactions and suggest at least 2 or 3 nucleotides may span the spacer region between the LBS-1 and LBS-2 sites (Figure 1F). Consistent with this finding, size exclusion chromatography demonstrates that mLANA_140–314_ forms tetramers in solution (Figure S2B). In addition, mLANA_140–272_ dimer forms a tetramer to cooperatively bind adjacent mTR DNA binding sites (described below).

**Figure 2:**
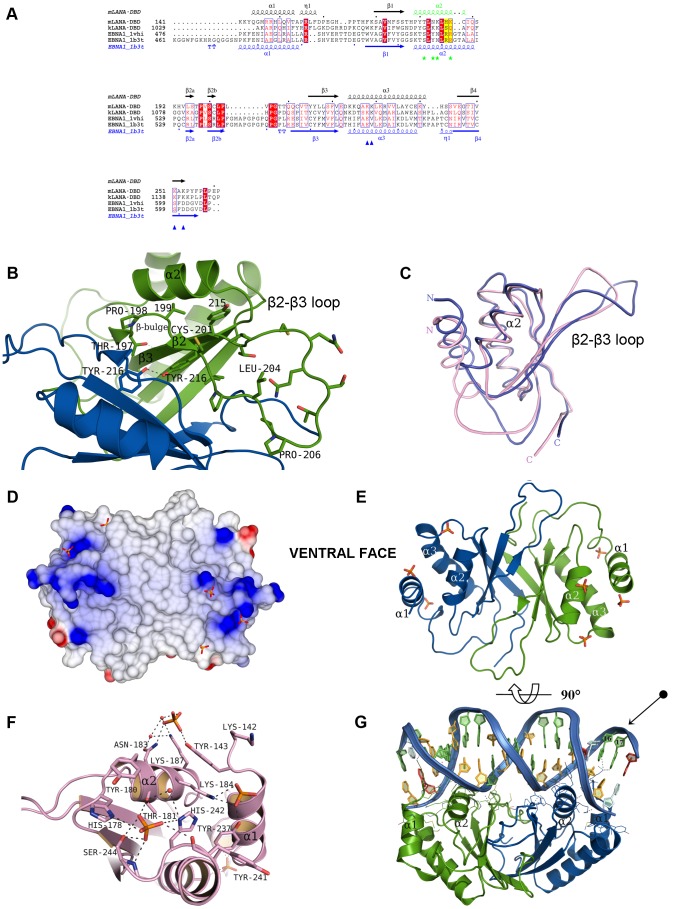
Conserved sequence and structural features of mLANA. (**A**) Structure based sequence alignment of the MHV-68 mLANA DBD with the DBD domains of KSHV kLANA, EBV EBNA1 (PDB ID: 1VHI), and EBNA1 DNA complex (PDB ID: 1B3T). The secondary structure of mLANA DBD is shown above the alignment while the secondary structure elements of EBNA1 structure are labelled below (blue). The recognition helix α2 is coloured in green with key residues highlighted. The recognition helix residues His-186 and Lys-187 are marked in yellow. Green stars indicate the residues of the recognition helix that interacted with phosphate ions. Conserved dorsal residues with positive charge are indicated by blue triangles. Figure is made with ESPript (http://espript.ibcp.fr/ESPript/ESPript/) Key: Red boxes and white characters indicate strict identity, red characters indicate similarity in a group and blue frames indicate similarity across groups. (**B**) Representation of the dimer interface on the ventral side highlighting the conformation of the β2–β3 SOCS box loop. The β2–β3 loop is the proposed hydrophobic interface for Elongin C interaction and shows the accessibility of residues Cys-201 and Leu-204 of the ^199^ VSCLPLVP ^206^ SOCS motif, shown as sticks. The interface highlights the conserved hydrogen bond between chain A Tyr-216 (blue) and chain B Tyr-216 (green) and shows the Pro-198 β-bulge. (**C**) Structural superposition of mLANA_140–272_ (chain B), in pink, with EBNA1 (PDB ID 1vhi; chain B), in blue (r.m.s deviation = 1.37 Å based on 82 equivalent Cα atoms). The recognition helix α2 in mLANA is shorter when compared to EBNA1 by 4 amino acids (1 helical turn), this has implications for both DNA binding and specificity. (**D**) mLANA dimer showing the electrostatic surface of the ventral side, the proposed DNA binding site and highlights five of the phosphate binding sites that trace DNA interactions. Electrostatic surface of mLANA was calculated and displayed using CCP4mg. The surface potentials displayed scale from −0.5 V (red, negatively charged) to +0.5 V (blue, positively charged). (**E**) A ribbon representation of the dimer in the same orientation, showing the secondary structure arrangement. (**F**) Representation of the recognition helix α2 and the key residues which interacts with 3 different phosphates through the side-chains of residues His-178, Thr-181, Asn-183, Lys-184 and Lys-187. (**G**) Structural model of a mLANA DBD DNA complex (rotated relative to panel E) using mLBS1 DNA docked onto the 18 bp EBNA1 DNA recognition sequence followed by energy minimisation using YASARA. The DNA shown was that which gave the best minimized score as indicated in Figure S3. In the structure each nucleotide is drawn in a different colour for clarity, adenine (red), guanine (green), cytosine (lightorange), and thymine (lightblue). The arrow indicates specific nucleotide interactions at sequence 16 and 17 of the modelled TR DNA.

Despite functional conservation, mLANA and EBNA1 proteins do not exhibit amino acid similarity (Figure 2A). However, a PHYRE2 program [40] search predicts mLANA secondary structure homology between the C-terminal domains of mLANA and EBNA1 (not shown), similar to other predictions [35]. A superposition of both monomer structures is shown in Figure 2C.

Protruding perpendicular to the ventral face of mLANA lies a partially disordered β2–β3 loop (199–215) (Figure 2B) that harbours a motif required for the interaction of mLANA with Elongin B/C (EloBC)-cullin-SOCS box (ECS)-type E3 ubiquitin ligase, resulting in the polyubiquitination of NFκB [31]. Mutations at the base of this loop (^199^ VSCLPLVP ^206^, underlined residues mutated to Alanine) disrupt binding of mLANA to Elongin C and consequently mLANA's E3-ubiquitin ligase activity. A recombinant virus containing these mutations fails to establish latency *in vivo* [31].

### Ventral face of mLANA – phosphate binding traces the site of DNA interaction

The structural basis for DNA recognition can be inferred from the observed phosphate binding pattern. In total, seven phosphates ions were located at the surface of mLANA. Five of these are situated on the ventral face (Figure 2D–F) while the remaining two cap the dorsal side of helix α1. Therefore, we consider the ventral face as the putative DNA binding site of mLANA. We modelled mLANA-DNA on the EBNA1-DNA structure (PDB ID: 1B3T) and the mLANA structure accommodated the DNA well (Figure 2G and Figure S3).

### Mutation of predicted DNA phosphate contacts disrupts DNA binding to TR DNA

**Figure 3:**
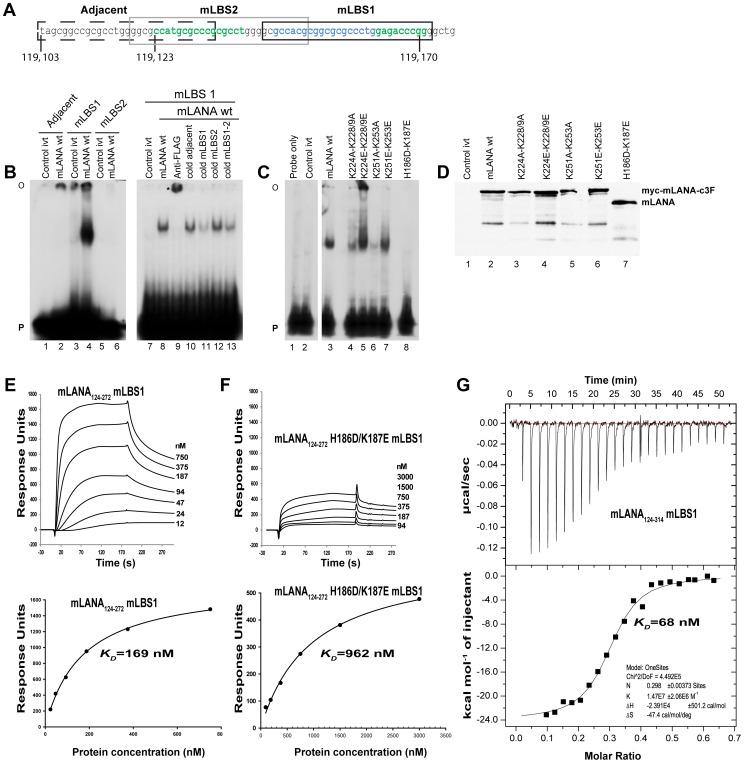
mLANA binds TR DNA with nanomolar affinity. (**A**) MHV-68 TR sequence with genomic coordinates is shown. Oligonucleotide sequences used for gel shift analyses are boxed. Sequence protected by DNAse I footprint analysis [24] is shown in blue and green. (**B**) mLBS1, mLBS2 or control adjacent ^32^P-labeled oligonucleotides were each incubated with *in vitro*-translated mLANAc3F wt or control reticulocyte lysate (control ivt). Anti-FLAG antibody, or 50 fold excess unlabeled (cold) competitor were included in incubations where indicated. FLAG antibody supershifted complex is indicated by an asterisk. (**C**) mLBS1 ^32^P-labeled oligonucleotide was incubated alone (lane 1), with control reticulocyte lysate (lane 2; control ivt), or the indicated *in vitro*-translated mLANA proteins. O, gel origin; P, free probe. (**D**) Western Blot is shown containing the same *in vitro*-translated mLANA proteins as in (C). (**E and F**) Panels show the SPR difference sensorgram with increasing concentrations of protein. Protein concentrations injected are as labelled. A 3-min association was followed by a 3-min dissociation phase. The bottom panels represent the *K*_D_ determination. The binding constant obtained by Langmuir fit of wild-type and mutant data sets using a 1∶1 stoichiometry-binding model. (**G**) ITC binding profile of mLANA_124–314_ with mLBS1 at 25°C. Top panel show raw differential power signals recorded versus time for 30 µM of mLBS1 titrations injected into a cell containing 8.5 µM of mLANA_124–314_ dimer. Bottom panel show integrated injection heats versus the molar ratio of mLANA to mLBS1. Kinetic parameters obtained from the experiments are given in Table 3.

To directly assess mLANA DNA binding, we used an electrophoretic mobility shift assay (EMSA) to assess the ability of mLANA to bind DNA. DNAse I footprinting analysis with recombinant mLANA has shown three adjacent protected regions within the mTR sequence [24], (Figure 3A, green and blue regions). Two overlapping probes, mLBS1 or mLBS2, each containing a portion of the protected sequence, or control, overlapping adjacent sequence were used to assess mLANA binding (Figure 3B). After incubation with mLANA, a shifted complex was only observed after incubation with mLBS1, and not with mLBS2 or control adjacent sequence (Figure 3B). The mLBS1 complex was supershifted with anti-FLAG antibody and was effectively competed with excess, unlabelled mLBS1 or mLBS1-2 competitor (containing the entire DNAse I protected region), but not unlabelled mLBS2 (Figure 3B). Despite the inability of mLANA to shift mLBS2 probe, size exclusion chromatography analysis demonstrated that mLANA_140–272_ bound adjacent mLBS1 and mLBS2 sites in mLBS1-2 oligonucleotide as a tetramer (Figure S6), consistent with cooperative binding to both sites. Therefore, similar to KSHV LANA DNA binding [10], mLANA cooperatively binds to adjacent high and low affinity TR DNA binding sites.

We then constructed mutations in the predicted mLANA recognition helix intended to decrease interactions with its cognate DNA. mLANA H186D/K187E contains substitutions predicted to disrupt phosphate interactions and abolish DNA binding, and this mutant was unable to complex with mLBS1 (Figure 3C). In contrast, mLANA K224A/K228A/K229A, mLANA K224E/K228E/K229E, mLANA K251A/K253A, and mLANA K251E/K253E, each contain substitutions within a dorsal positively charged patch (discussed below) opposite the predicted DNA interface, and each mLANA gel shifted the mLBS1 probe (Figure 3C). The mLANA K251E/K253E (Figure 3C, lane 7) complex was similar in intensity to that of mLANA (Figure 3C, lane 3), though the intensities of the mLANA K224A/K228/K229A and mLANA K251A/K253A complexes were each reduced (Figure 3C, comparing lane 4 and 6 with lane 3). The reduced mLANA K224A/K228A/K229A and mLANA K251A/K253A binding was likely in part due to modestly lower protein expression (Figure 3D). Notably, mLANA K224E/K228E/K229E demonstrated increased mLBS1 binding (comparing lane 5 with lane 3). Therefore, as predicted by the mLANA DNA binding domain structure, mLANA H186D/K187E substitutions abolished DNA binding, but DNA binding was preserved with dorsal mutations. The finding that dorsal alanine substitutions of lysine reduced DNA binding, while glutamate substitutions did not, suggests that substituting non charged residues may have subtle effects on DNA binding domain folding.

We characterised mLANA TR DNA binding by surface plasmon resonance (SPR) and isothermal titration calorimetry (ITC) to determine the dissociation constant (*K*_D_) for mLBS1. The *K*_D_ for wild type and H186D/K187E were determined by titrating mLANA_124–272_ against immobilized mLBS1 (Figure 3E and F). The *K*_D_'s were 169 nM and 960 nM for wild type and mLANA H186D/K187E, respectively (Table 2). Thermodynamics binding parameters in solution measured by ITC using the more soluble mLANA_124–314_ fitted well to a one-site model, indicating one distinct mLANA binding site (Figure 3G), and provided a calculated *K*_D_ of 66 nM (Table 3). The discrepancy between the SPR and ITC dissociation constants was due to the inaccessibility of more than 50 percent of mLANA_124–314_ for DNA binding as observed from the calculated N value from ITC (0.27±0.03 dimer per TR-DNA)(Supporting Information, Text S1).

**Table 2:** Interaction of mLANA_124–272_ and mLANA_124–272_ H186D/K187E with TR-DNA.

K_D_	WT	Mut H186D/K187E
**EMSA** *	Binding	No binding
**SPR**	169 nM	960 nM
**ITC**	66±12 nM	ND

* Full-length mLANA protein used in this experiment.

**Table 3:** Kinetic values for mLANA_124–314_ with TR-DNA by ITC.

	ΔH^0^ kJ/mol	−ΔTS^0^ kJ/mol	ΔG^0^ kJ/mol	K_D_ nM	n
**WT**	−103.9±3.4	63.0±3.4	−41.0±0.5	66±12	0.27±0.03

Standard deviations were calculated from three independent measurements. Gibbs free energy and entropy were calculated using these equations ΔG = RT lnK_D_ and ΔG = ΔH−TΔS.

### mLANA exhibits a novel positive patch on the dorsal face and interacts with BRD4

**Figure 4:**
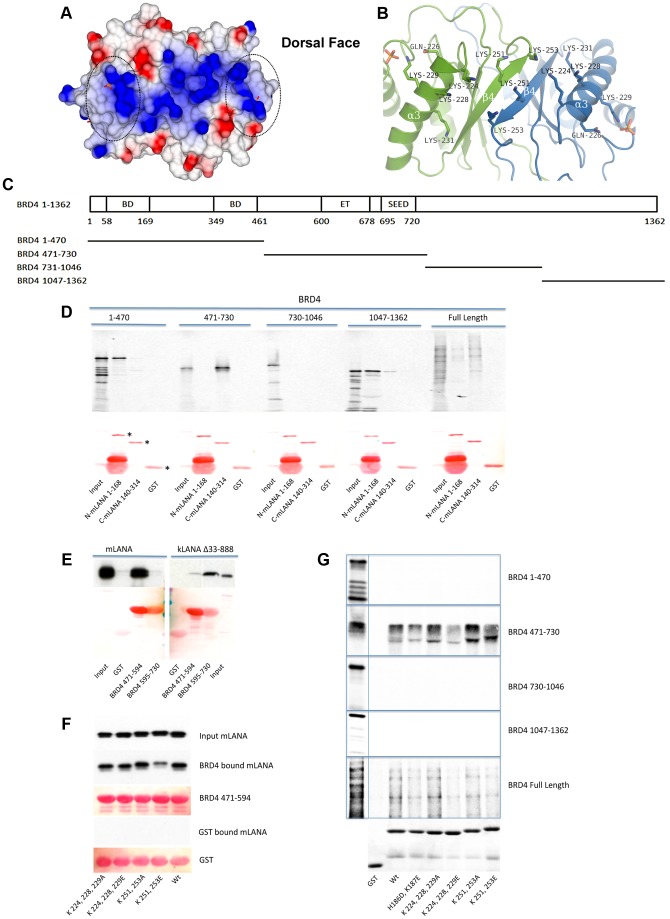
The dorsal face of mLANA and its role in BRD4 binding. (**A**) Electrostatic surface showing the residues that contribute to the positive electrostatic potential, Arg-156, Lys-224, 225, 228, 229, 231, and Arg-232, which run along the spine of the dorsal face. The QAKKLK motif is highlighted. The scale is from −0.5 V (red, negatively charged) to +0.5 V (blue, positively charged). (**B**) Ribbon diagram highlighting the key positively charged residues. (**C**) Schematic diagram of BRD4 and fragments used for mapping the mLANA binding. BD, bromodomain; ET, extra-terminal domain; SEED, conserved region consists of polyserine (S) residues interspersed with glutamic (E) and aspartic (D) acid residues [62], [63]. (**D**) BRD4 fragments were labelled with [^35^S]-methionine and assayed for binding to GST mLANA 1–168 or GST mLANA 140–314. Ponceau S detection of proteins (lower panel) is shown and full length GST, GST mLANA 1–168, or GST mLANA 140–314 are indicated with asterisks. (**E**) Western blots for mLANA and kLANA are shown in the upper panel; the Ponceau S stained GST and GST fusion proteins are displayed in the lower panel. Despite the lower intensity of the GST and GST BRD4 595–730 bands after Ponceau S staining, Coomassie blue staining (Figure S4) showed that similar amounts of fusion proteins were present. (**F**) After incubation with GST or GST BRD4 471–594, bound mLANA was detected by FLAG immunoblot. Ponceau S staining of fusion proteins is shown. (G) BRD4 or BRD4 fragments labelled with [^35^S]-methionine were assayed for binding to GST mLANA 124–272 or GST mLANA 124–272 containing substitution mutations. Coomassie blue staining of fusion proteins is shown in the bottom panel.

Electrostatic surface analysis of the mLANA protein reveals an extensive positive patch on the dorsal side which includes residues Arg-156, Lys-224, 225, 228, 229, 231, 251, 253, and Arg-232 (Figure 4A and B and S5B). Both Arg-156 and Lys-229 interact with a phosphate ion which is located on the dorsal face of helix α1. Lys-251 and 253 lie on the 2-fold non-crystallographic symmetry axis of the dimer interface (Figure 4B). The positive electrostatic surface also encompasses the identified ^226^QAKKLK^231^ motif that is involved in binding to BET proteins [34]. Both mLANA and kLANA have been described to bind to the ET domain of BET proteins and the cluster of positive charges is energetically organized to establish charge-charge interactions with a negatively charged patch on the helical ET domain of the BET proteins BRD2 and BRD4. Four key Lysine residues are conserved in both mLANA (Lys-228, Lys-229, Lys-251, and Lys-253) and kLANA (Lys-1113, Lys-1114, Lys-1138 and Lys-1140) (Figure 2A).

Although mLANA was previously described to bind BRD4, a complete mapping of mLANA binding within BRD4 had not been performed. Therefore, we investigated mLANA binding to BRD4 (Figure 4C). GST mLANA 1–168 or GST mLANA 140–314 were used to assess binding to *in vitro* translated BRD4 or fragments spanning full length BRD4. GST mLANA_140–314_ precipitated full length BRD4 more efficiently than did GST mLANA_1–168_ (Figure 4D). Interestingly GST LANA mLANA_1–168_ bound BRD4 at two sites, BRD4 1–470 and BRD4 1047–1362, while GST mLANA 140–314 bound BRD4 471–730 (Figure 4D). Since BRD4 471–730 contains the conserved ET domain in its C-terminal portion, we then assessed binding of mLANA to GST BRD4 471–594 (upstream of the ET domain) or GST BRD4 595–730 (ET domain and SEED motif). mLANA bound BRD4 471–594, upstream of the ET domain while consistent with previous results [41], kLANA bound the ET domain containing region in BRD4 595–730 (Figure 4E).

Since mLANA residues 226–231 were previously shown to be important for BRD4 binding [34], and since C-terminal mLANA bound the region upstream of the ET domain, we assessed the ability of mLANA, or mLANA mutated at positively charged residues, including within 226–331, to bind BRD4 471–594. mLANA K251E/K253E and mLANA K251A/K253A contain either negatively charged or neutral substitutions at residues in the central positive patch region, while mLANA K224E/K228E/K229E and mLANA K224A/K228A/K229A contain similar substitutions at the periphery of the positive patch. mLANA and each of the mutants bound BRD4 471–594 (Figure 4F). However, BRD4 binding was substantially reduced for mLANA K251E/K253E and modestly reduced for mLANA K224E/K228E/K229E (Figure 4F). In addition, we assessed the ability of GST mLANA 124–272, GST mLANA 124–272 mutated at dorsal positively charged residues, or GST mLANA 124–272 H186D/K187E, which is abolished for DNA binding (Figure 3), for the ability to bind BRD4 or fragments spanning BRD4. GST mLANA 124–272 bound BRD4 and BRD4 471–730 (Figure 4G), similar to GST mLANA 140–314 (Figure. 4D). Each of the mLANA mutants also bound both BRD4 and BRD4 471–730, although binding was reduced for mLANA 124–272 H186D/K187E, mLANA K224E/K228E/K229E, and mLANA K251E/K253E (Figure 4G). The finding that mLANA 124–272 H186D/K187E had reduced BRD4 binding suggests a potential role for these residues in interacting with Brd4. Therefore, substitution of negatively charged, but not neutral, residues for positively charged amino acids on the dorsal positive patch of C-terminal mLANA reduces BRD4 binding.

### Loss of DNA binding abolishes virus persistence in mice

**Figure 5:**
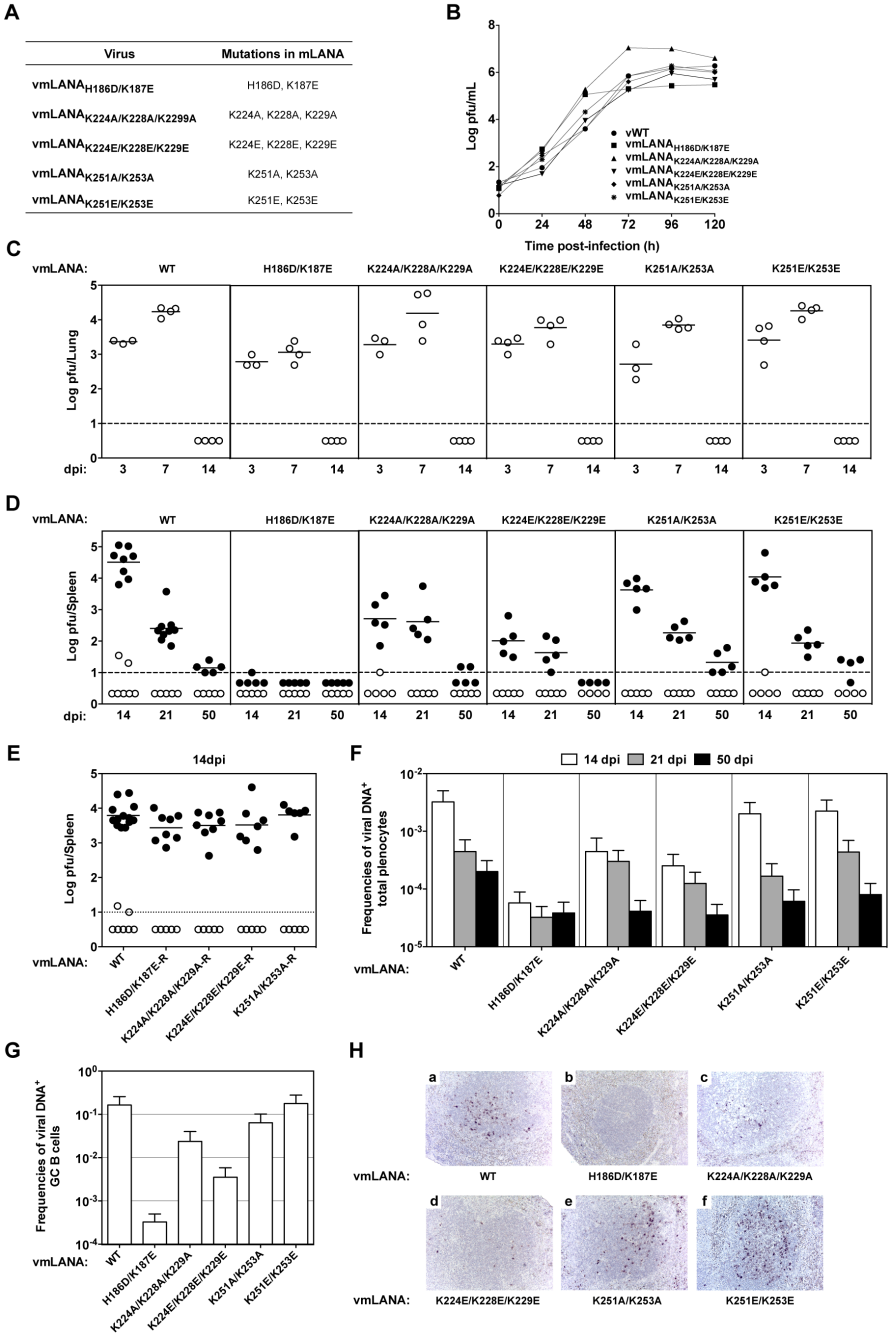
mLANA DNA binding is essential for virus persistence and the dorsal positive patch exerts a role in the expansion of GC B cells. (**A**) Amino acid substitutions in recombinant viruses (see also Figure S5). (**B**) Infection of BHK-21 cells at 0.01 p.f.u. per cell. Virus titres were determined by plaque assay. (**C**) Lungs from infected mice were removed and infectious viruses were titrated by plaque assay. (**D and E**) Quantification of latent infection in spleen by explant co-culture plaque assay (closed circles). Titres of infectious virus were determined in freeze/thawed splenocyte suspensions (open circles). Each circle represents the titre of an individual mouse. The dashed line represents the limit of detection of the assay. Mutant viruses are shown in panel D and revertant viruses in panel E. (**F and G**) Reciprocal frequencies of viral DNA-positive cells in total splenocytes (**F**) or GC B cells (CD19^+^CD95^hi^GL7^hi^) (**G**) were determined by limiting dilution and real-time PCR. Data were obtained from pools of five spleens per group. Bars represent the frequency of viral DNA-positive cells with 95% confidence intervals. (**H**) Identification of latently infected cells in spleens by *in situ* hybridization. Representative splenic sections from each group of viruses are shown. All images are magnified ×200. Dark staining indicates cells positive for virally encoded miRNAs (see also Table 4).

We assessed the role of mLANA binding to viral DNA on virus persistence in mice. A recombinant virus was generated bearing the mLANA H186D/K187E mutations in the predicted mLANA DNA recognition helix (designated vmLANA_H186D/K187E_; Figure 5A). We first compared the kinetics of lytic viral replication *in vitro* and during the acute phase of infection in lungs. Lytic replication kinetics was essentially preserved, though changes in the pattern of lytic infection could be identified (Figure 5B and C; Supporting Information, Text S1). This result is consistent with mLANA having a predominant role in latency. We next proceeded to investigate the impact of the introduced mutations in the ability of MHV-68 to expand latent infection in GC B cells and establish persistent infection in mice. vmLANA_H186D/K187E_ showed a marked defect in *in vitro* reactivation from latency (Figure 5D), and viable virus was barely detectable beyond the limit of detection of the assay as early as 14 dpi. This viral mutant also showed a clear defect in the frequency of viral-DNA positive splenocytes (Figure 5E and Table 4). Consistent with this deficit, the frequency of vmLANA_H186D/K187E_ infection in GC B cells at the peak of virus latency (14 dpi) was three orders of magnitude lower (Figure 5F and Table 4). This result was confirmed by visualisation of latently infected cells within GCs in splenic sections (Figure 5G, a–b). Notably, analysis of vmLANA_H186D/K187E_ at 21 and 50 dpi showed that loss of mLANA DNA binding severely diminished virus persistence in mice (Figure 5D and E). These results demonstrate that mLANA binding to its cognate DNA is essential for the expansion of latent infection in GC B cells and is critical for virus persistence.

**Table 4:** Reciprocal frequency of MuHV-4 infection in total splenocytes and GC B-cells^a^.

Cell subpopulation	Dpi	Virus	Reciprocal frequency^b^ of viral DNA positive cells	
Total splenocytes	14	vWT	310	(198–707)
		vmLANA_H186D/K187E_	17377	(11229–38406)
		vmLANA_K224A/K228A/K229A_	2242	(1311–7712)
		vmLANA_K224E/K228E/K229E_	3953	(2502–9373)
		vmLANA_K251A/K253A_	499	(317–1170)
		vmLANA_K251E/K253E_	448	(287–1019)
	21	vWT	2243	(1405–5545)
		vmLANA_H186D/K187E_	30949	(20094–67314)
		vmLANA_K224A/K228A/K229A_	3322	(2148–7334)
		vmLANA_K224E/K228A/K229E_	7985	(5134–17955)
		vmLANA_K251A/K253A_	5979	(3653–16461)
		vmLANA_K251E/K253E_	2296	(1442–5630)
	50	vWT	4976	(3220–10952)
		vmLANA_H186D/K187E_	24175	(15796–51478)
		vmLANA_K224A/K228A/K229A_	28081	(18461–58638)
		vmLANA_K224E/K228E/K229E_	16282	(10332–38380)
		vmLANA_K251A/K253A_	12444	(8010–27880)
GC B-cells^c^	14	vWT	6	(4–14)
		vmLANA_H186D/K187E_	3079	(2006–6619)
		vmLANA_K224A/K228A/K229A_	30	(20–59)
		vmLANA_K224E/K228E/K229E_	284	(171–841)
		vmLANA_K251A/K253A_	16	(10–39)
		vmLANA_K251E/K253E_	6	(4–13)

a Data were obtained from pools of at least five spleens. WT, wild type.

b Frequencies were calculated by limiting-dilution analysis with 95% confidence intervals (numbers in parentheses).

c The purity of sorted cells was determined by fluorescence-activated cell sorting (FACS) analysis, and was always greater 95%.

### Electrostatic surface residues on the dorsal face exert a role in expansion of latent infection in GC B cells

We investigated the role of the positive electrostatic surface residues on the pathogenesis of MHV-68 infection taking into account their charge and relative position on the dorsal face. Recombinant viruses were engineered to contain neutral or negatively charged mutations at the centre or periphery of the positive patch (Figure 5A). This set of mutations did not affect viral replication *in vitro* or affect lytic infection in lungs of mice (Figure 5B and C). However, when the latent phase of the infection was analysed a clear phenotype could be ascribed to mutations located at the periphery of the positively charged dorsal motif. Whereas vmLANA_K251A/K253A_ and vmLANA_K251E/K253E_ showed levels of latency comparable to wild type virus (Figure 5D, F and Table 4), viruses vmLANA_K224A/K228A/K229A_ and vmLANA_K224E/K228E/K229E_ showed a significant (p<0.0001) latency deficit at 14 dpi (Figure 5D). This deficit was confirmed by quantification of the frequency of viral DNA-positive cells in total splenocytes (Figure 5F and Table 4). This phenotype is unlikely to reflect inadvertant mutations introduced during the mutagenesis process since the equivalent phenotype was observed in both independently derived mutant viruses. Moreover, revertant viruses in which the mutated K224/K228/K229 residues were restored to wild type status did not show any defects in latent infection (Figure 5E), demonstrating that phenotypic changes observed were intrinsic to this locus and not a consequence of mutations elsewhere in the viral genome. Quantification of the frequency of infection in purified GC B cells (Figure 5G and Table 4) and visualisation of latently infected cells by *in situ* hybridisation (Figure 5H, c–d) revealed that replacing a positively charged lysine for the oppositely charged glutamate accentuated the attenuated phenotype when compared to replacement with a non-polar alanine. Notably, the attenuated latency phenotype ascribed to Lys-224, 228, 229 did not correlate with the activity of mLANA to bind BRD4. That conclusion is based on the result that BRD4 binding was reduced for mLANA K251E/K253E (Figure 4F and 4G), however vmLANA_K251E/K253E_ showed no latent infection phenotype (Figure 5D, F, G and H, e–f). Further, mLANA K224A/K228A/K229A bound BRD4 normally (Figure 4F and 4G), yet vmLANA_K224A/K228A/K229A_ was deficient for latent infection. Taken together these data demonstrate that Lys residues 224, 228 and 229 located at the periphery of the positively charged dorsal motif are required for the efficient expansion of infected GC B cells, likely acting through a host cell protein other than BRD4.

## Discussion

C-terminal LANA is the most highly conserved LANA domain, and its ability to bind DNA is essential for viral DNA replication and episome persistence [12]. Here, we solve the X-ray crystal structure of the mLANA DNA binding domain. The overall fold comprises a rigid and thermostable β-barrel dimer flanked on either side by an α-helix arrangement. The structural features of mLANA are common to EBV EBNA1 and HPV E2 proteins, indicative of their conserved biological functions in episome maintenance and transcriptional activation.

This work identifies ventral C-terminal mLANA as the DNA binding interface and demonstrates its essential role in latent viral infection. The similarity in the protein folds of mLANA with EBNA1, the sites of mLANA phosphate ion binding, and the modelled superposition of the EBNA1-DNA complex all support that the ventral side interfaces with DNA. Furthermore, a high affinity TR DNA LANA binding site (mLBS1) was demonstrated by EMSA and shown by ITC to bind mLBS1 with nanomolar affinity, 66 nM, with enthalpy as the major driving force for binding. Similar to kLANA, mLANA cooperatively binds adjacent high and low affinity TR DNA binding sites. Disruption of targeted residues on the ventral surface abolished DNA binding (Figure 3), providing direct evidence that this surface is the DNA binding interface. Moreover, engineering the same disruptive mutations into the genome of MHV-68 abolished the ability of the virus to expand latent infection in GC B cells and eliminated persistence of virus that was capable of reactivation. This result was consistent with recent findings that virus containing mLANA incapable of binding DNA was highly deficient for virus *ex vivo* reactivation from splenocytes [24]. This work establishes that LANA binding to TR DNA is critical for viral persistence in proliferating GC B cells. Most importantly, it supports a model in which a strategy of expansion of virus infection in GC B cells to access persistence in long-lived memory B cells is vital for host colonisation. This establishes a parallel with EBV where the same strategy for virus persistence has been proposed based upon clinical investigation [7].

Prior genetic and functional analyses of the LANA DBD can now be interpreted in light of the solved mLANA structure. Consistent with their predicted contacts with DNA, mutations within α helices 1 and 2 affect DNA binding. mLANA Pro179Thr or Leu150Pro resulted in loss of the ability of mLANA to protect its binding site from DNase I cleavage [24], [39], [42]. Pro-179 is found at the N-terminus turn of the α2-helix, and restricts the dihedral angles of the preceding amino acid His-178, diminishing the flexibility of His-178, a residue expected to be important for DNA interaction (Figure 2F). The Leu150Pro substitution in helix α1 causes helix disruption as proline is expected to destabilize the α-helix structure, causing a kink. Multiple mutations within α helices 1 and 2 of the kLANA DBD also disrupted DNA binding [24], [39], [42]. As some kLANA mutations located in regions other than α helix 1 and 2 also disrupted binding, it is likely that these disturb secondary structure and folding of the DBD. Consistent with the large surface area interface of the dimer, only larger deletions of ∼10–15 amino acids were able to disrupt kLANA self-association [12], [39], [43].

Inhibition of NF-κB signalling is essential for MHV-68 latency [31]. mLANA recruits EC_5_S E3 ubiquitin ligase complex to target NFκB for degradation by the ubiquitin-proteasome pathway by way of an unconventional SOCS box motif (^199^ **V**SC**LP**LV**P^206^**). SOCS box containing proteins mediate interactions with elongins B and C to target substrates for degradation. The mLANA structure shows that this motif forms a loop perpendicular to the DNA binding interface, likely allowing accessibility for elongin binding without disrupting DNA binding. A strictly conserved leucine residue [44], [45] is part of an essential hydrophobic interface required for Elongin C binding. This feature may be fulfilled by residues Leu-202, Pro-203 and/or Leu-204 that are positioned on the β2–β3 loop to accommodate an ElonginBC-Cul5 complex. A conserved cysteine is critical for SOCS-box interaction with Elongin C [44], and the LANA SOCS-box motif includes Cys-201 conserved in both mLANA and kLANA. The mLANA SOCS loop is an atypical structural motif since a typical SOCS-Elongin C interaction forms a “folded-leaf” four helix cluster. Since LANA does not display a standard SOCS box consensus sequence, it is likely that LANA has acquired a unique strategy to recruit an EC_5_S ubiquitin ligase complex.

The most striking feature in the mLANA structure is the positive electrostatic charge on the dorsal face, which is absent in EBNA1 and E2. Residues ^226^QAKKLK^231^, within this charged region, were previously identified as binding the BET family of proteins, BRD2 and BRD4 by using BRD4 as a probe against an mLANA peptide array and these findings were confirmed in co-immunoprecipitation experiments with mLANA mutated at these residues [34]. Consistent with these results, we found that substitution of positively charged for negative residues in mLANA K224E/K228E/K229E reduced BRD4 binding, although substitution with neutral residues in mLANA K224A/K228A/K229A did not. Similarly, mLANA K251E/K253E, but not mLANA K251A/K251A, was reduced for BRD4 binding. These findings suggest possible electrostatic inhibition when positively charged residues were substituted for ones with a negative charge. Our results differ from other work [34] since the region immediately upstream of the BRD4 ET domain, rather than the ET domain itself, mediated C-terminal mLANA binding. Interestingly, in contrast to mLANA, and as previously reported [41], kLANA bound the ET domain rather than the upstream BRD4 region, suggesting independent evolution of this LANA binding. We also found that N-terminal mLANA binds to distinct BRD4 regions compared to C-terminal mLANA, although the C-terminal domain has the more prominent role in BRD4 binding (Figure 4). Interestingly EBNA1 and E2 each bind BRD4 through non C-terminal regions. EBNA1 binds BRD4 through N-terminal residues 61–83 [46], while the N-terminal E2 transactivation domain binds BRD2/4 [47]. Most importantly, our mouse infections with mutated viruses showed that this novel electrostatic charge patch on the mLANA dorsal face is required for efficient expansion of latently infected GC B cells. The charged patch could be divided into two components with different functional effects, consisting of a central region and a peripheral region. Unexpectedly, the attenuated latency phenotype ascribed to mutations in Lys-224, 228, 229 could not be attributed to BRD4. This finding suggests that this region is likely to be acting through a host cell protein other than BRD4, possibly a different BET protein.

The resolution of the crystal structure of the DBD of mLANA provides advantages for pathogenesis studies in a mouse model. In this study, solving the mLANA DBD structure enabled the rational design of mutations in the DNA binding interface that resulted in loss of DNA binding and eliminated virus associated GC cell proliferation and persistence of viable virus in the host. In addition, the mLANA quaternary structure revealed a novel structural motif composed of a patch of positively charged lysine residues on the dorsal face. Results showed that this feature exerts a key role in the expansion of latently infected GC B cells. Thus, combining structural, in vitro and cellular studies with an animal model of infection offers a unique opportunity to investigate viral pathogenesis.

## Materials and Methods

### Ethics statement

This study was carried out in strict accordance with the recommendations of the Portuguese official Veterinary Directorate (Portaria 1005/92). The Portuguese Experiments on Animal Act strictly comply with the European Guideline 86/609/EEC and follow the FELASA. Animal experiments were approved by the Portuguese official veterinary department for welfare licensing under the protocol number AEC_2010_017_PS_Rdt_General and the IMM Animal Ethics Committee.

### Cloning, expression and purification of mLANA

A series of C-terminal truncations of mLANA DBD were cloned into the pET-49b vector (Novagen) and the proteins expressed with an N-terminal GST-His-tag (Figure S1) in *Escherichia coli* BL21 Star (DE3) cells (Invitrogen) containing the pRARE2 plasmid (Novagen). Proteins were purified on an ÄKTA Explorer (GE Healthcare) FPLC system. Point mutations within mLANA were engineered using the QuikChange multisite-directed mutagenesis kit (Stratagene). Truncations of mLANA (Figure S1A) were PCR cloned into pET-49b as BamHI-NotI fragments using the appropriate primers (Figure S1B). All constructs were confirmed by DNA sequencing. The vector carries an N-terminal GST-tag and His-tag coding sequences followed by a recognition site for the human rhinovirus (HRV) 3C protease (LEVLFQ/GP). Cultures of BL21 Star (DE3) cells transformed with mLANA plasmids were grown until the mid-log phase (A600 nm = 1.8) in Terrific Broth medium supplemented with kanamycin (50 µg/ml) and chloramphenicol (30 µg/ml), induced with 0.5 mM isopropyl-1-thio-β-D-galactopyranoside, and grown for an additional 16 h at 18°C. Cells were harvested by centrifugation, resuspended in a buffer containing 50 mM Na/K phosphate (pH 7.0), 500 mM NaCl, 1 mM Tris (2-carboxyethyl) phosphine (TCEP), 10 mM imidazole, EDTA-free protease inhibitor cocktail (Roche) and 5 µg/ml OmniCleave, (Epicentre), and stored at −80°C. The cell pellet was disrupted using an APV-2000 continuous homogenizer (APV, Madrid, Spain) at 6000 psi. After centrifugation, the supernatant was loaded onto a HisTrap HP (GE Healthcare) column, pre-equilibrated in 50 mM Na/K phosphate (pH 7.0), 500 mM NaCl, 1 mM TCEP, 10 mM imidazole, and proteins eluted with a linear gradient from 10–500 mM imidazole in the same buffer using a ÄKTA Explorer (GE Healthcare) FPLC system (Figure S1C). The GST-Histidine tag was removed by overnight incubation with 3C protease at 4°C and the cleaved protein passed over a GSTPrep FF (GE Healthcare) column (to remove any uncleaved protein as well as the GST-tagged 3C protease) connected end to end to a HiPrep Heparin FF (GE Healthcare) column. The columns were disconnected and the heparin bound mLANA proteins eluted with a linear gradient from 0.3–2 M NaCl. Following 3C cleavage the recombinant protein contained the vector-derived sequence GPGYQKDP at the NH2-terminus. Finally, the protein was applied to a Superdex S75 16/60 filtration column (GE Healthcare) in 10 mM MES buffer, pH 6.5, 300 mM NaCl and 1 mM TCEP and stored at −80°C.

### Differential scanning fluorimetry

Assays were performed on an iCycle IQ5 Real Time PCR Detection System (BioRad) with excitation and emission wavelengths of 490 and 575 nm, respectively. Reaction volumes of 20 µl contained 1 µg of protein and 10× of SYPRO orange (Invitrogen) previously diluted in 20 mM HEPES pH 7.5 from an initial 5000× stock. The reaction mix was prepared by adding 2.0 µl of protein-dye mixture solution to 18.0 µl of each buffer from the JBS Solubility kit (Jena Biosciences). For the thermal denaturation the plates were heated at a rate of 1°C/min from 20 to 90°C with a 10 s hold step for every point and fluorescence was measured in 1°C increments. Experiments were carried out in duplicate, and T_m_ values were calculated for each well using nonlinear regression analysis in the curve-fitting program GraphPad Prism. The T_m_ values were incorporated into a Microsoft Excel script (ftp://ftp.sgc.ox.ac.uk/pub/biophysics) for comparison of melting curves and thermal shifts.

### Crystallization and structure determination

Screening of crystallization conditions was performed using four sparse matrix formulations from commercial screens (Structure Screen I+II, PACT-premier, JCSG-plus and Stura Footprint combination from Molecular Dimensions, UK) in round-bottom Greiner 96-well CrystalQuick plates (Greiner Bio-One) and performed using a Cartesian Crystallization Robot Dispensing System (Genomics Solutions). The sitting-drop vapour-diffusion method was used, with 100 nl protein sample (30 mg/ml) mixed with an equal volume of the reservoir screening solution. In less than 1 day at 21°C, mLANA_140–261_ crystallized as small needle shaped crystals in condition E10 (0.1 M Tris-HCl pH 8.5, 20% w/v PEG 2000 MME, 0.01 M nickel chloride) of the Structure Screen and truncation mLANA_140–272_ crystallized as thin, but large, plate-like crystals in condition F10 (0.1 M Tris-HCl pH 8.5, 25% w/v PEG 3350, 0.2 M lithium sulphate) of the Stura Footprint Combination Screen.

While crystal optimization by sitting-drop vapour diffusion technique failed for mLANA_140–261_. Diffraction-quality crystals for mLANA_140–272_ were obtained by exchanging the protein buffer to the optimal buffer system suggested by DSF assays (50 mM Na/K phosphate pH 7.0). mLANA_140–272_ crystals were further optimized by serial streak-seeding in decreasing precipitant concentrations, with optimal growth to 0.05×0.05×0.15 mm in drops formed by mixing an equal volume of the protein solution with a reservoir solution that contained 0.1 M Na/K phosphate pH 7.0, 0.1 M lithium sulphate, 22% w/v PEG 3350 and 4% v/v 1,4 dioxane. Crystals were transferred to mother liquor supplemented with 20% v/v glycerol, and cryocooled in liquid nitrogen.

A diffraction data set was collected from EMBL beamline P14 at PETRA III (DESY, Germany), indexed and integrated with XDS [48], then merged and converted with Pointless [49] to mtz format for scaling in SCALA [49]. The structure was solved by molecular replacement using Phaser [50] with the EBNA1 dimer (PDB ID: 1VHI) as a search model. An initial model of the structure was autobuilt in PHENIX [51] with manual rebuilding of the model performed in Coot [52] and refined in PHENIX. The crystallographic data are summarized in Table 1. The structure was validated using MOLPROBITY [53] as implemented in PHENIX. The analysis of the dimer interface used the Protein interfaces, surfaces and assemblies service PISA at European Bioinformatics Institute [54]. Energy minimization for 3D structures of mLANA-DNA models was performed using YASARA [55]. Structural illustrations were prepared with PyMOL [56] and CCP4MG [57].

### Electrophoretic mobility shift assays

mLANA WT and mutants were in vitro-translated using TNT coupled Reticulocyte Systems (Promega # L4610) from pBS-mLANAc3F WT (Figure 3B), pBS mLANA H186D/K187E, pBS myc mLANAc3F wt (Figure 3C) and pBS mLANA K224A/K228/K229A, pBS mLANA K224E/K228/K229E, pBS mLANA K251A/K253A, or pBS mLANA K251E/K253E. mLBS probes were ^32^P-labeled using Prime-It II (Agilent technology #300385). EMSAs were performed using 20,000 (panel B) or 30,000 (Figure 3C) cpm ^32^P-labeled mLBS1, mLBS2, or control adjacent sequence, each also containing a GATC 5′ overhang. Probes were incubated 20 min. at RT with 5 to 10 µL of in vitro translated mLANA in EMSA buffer (20 mM Tris-HCl pH 7.5, 50 mM KCl, 10 mM MgCl2, 1 mM EDTA, 20 µg/mL polydI-dC [Amersham Bioscience #27-7880-02], 0.1 mM DTT and 10% glycerol). For competition controls, 50 fold excess unlabeled mLBS1, mLBS2, or mLBS1-2 were included in the incubation. To generate mLBS1-2 oligonucleotide, TATAATGGATCCggcgccatgcgcccgcgcctggggcgccacgcggcg and TTATATGGATccccgggtctccagggcgcgc-cgcgtggcgccccag primers were annealed and extended using Prime-It II kit (capital letters indicate additional non TR sequence). The resulting oligonucleotide was ligated into the EcoRV site of pBluescript. mLBS1-2 was then released after digestion with HindIII/XbaI and gel purified (Qiagen #28406). For the supershift, 1 µg anti-FLAG antibody (Sigma #F1804) was added to the reaction. Loading buffer (95% Formamide, 10 mM EDTA, 0.1% xylene cyanol and 0.1% bromophenol blue) was added to incubations prior to loading on a 3.5%/8% non-denaturing TBE-polyacrylamide gel. The gel was run in TBE buffer for 1 hour at 300 V and dried on Whatman paper before exposing to Kodak biomax MS film (Kodak #829 4985). *In vitro* translated mLANA wt and mutants were detected by western blot. mLANA was resolved by 10% SDS-PAGE and after transfer to nitrocellulose membrane, the protein detected using monoclonal anti-mLANA (6A3) followed by HRP conjugated goat anti-mouse secondary antibody (Southern Biotech #1031-05).

### SPR and ITC analysis

SPR binding experiments were performed on a BIACORE ×100 instrument (GE Healthcare) at 25°C. Single stranded DNA oligonucleotides, mLBS1 with an addition of five thymine's in the 5′ end as a spacer were annealed to its pair before injected over a streptavidin-coated sensor chip (SA chip, GE Healthcare) at 1 nM concentration at 10 µl min^−1^ up to 590 RU. A flow cell was left blank to allow background signal subtraction.

Purified recombinant mLANA_124–272_ protein dilution series from higher to lower concentration were injected at a flow rate of 10–30 µL min^−1^ for 3 min and the regeneration of the binding surface was achieved by 4M NaCl. All the experiments were performed in duplicates. Data were processed using BiaEvaluation (GE Healthcare) and analyzed using SigmaPlot. The equilibrium dissociation constant, *K_D_*, was determined by non-linear fitting of the data using a 1∶1 Langmuir isotherm.

We further examined mLANA-DNA binding using ITC. Purified mLANA protein_124–314_ and mLBS1 DNA (without biotin-TTTTT spacer) were dialyzed against 25 mM Na/K phosphate pH 7.0, 150 mM NaCl, and 5% glycerol. ITC titrations were performed with a MicroCal iTC200 Isothermal Titration Calorimeter (MicroCal) at 25°C. Protein and DNA absorbance were measured after dialysis by NanoDrop (NanoDrop Technologies) and their concentrations were determined with their respective extinction coefficients. Twenty-five injections of 1.5 µl each of 30 µM mLBS1 DNA were titrated into 8.5 µM protein solution. Data were corrected for nonspecific heats and analysed using MicroCal Origin 7.0 software using a one-site binding model. The experiments were performed in triplicate and showed similar results.

### In vitro binding of mLANA and BRD4

BRD4, BRD4 1–470, 471–730, 731–1046, or 1047–1362 [41] were *in vitro* translated and labelled with [^35^S]-methionine using TNT Coupled Reticulocyte Lysate System (Promega). mLANA K224A/K228A/K229A, mLANA K224E/K228E/K229E, mLANA K251A/K253A or mLANA K251E/K253E each were engineered to include three tandem C-terminal FLAG epitope tags and were in vitro translated from pBluescript. GST, GST-N-mLANA_1–168_ or GST-C-mLANA_140–314_ were expressed from pGEX-2T. GST mLANA 124–272, GST mLANA 124–272 H186D/K187E, GST mLANA 124–272 K224A/K228A/K229A, GST mLANA 124–272 K224E/K228E/K229E, GST mLANA 124–272 K251A/K253A and GST mLANA 124–272 K251E/K253E fusion proteins were expressed from pET-49. GST, GST mLANA or GST BRD4 [41] fusion proteins were expressed in BL21 (DE3) bacteria and collected on Glutathione Sepharose 4B (GE Healthcare) beads. GST fusion binding assays were performed as described [41]. Proteins were resolved by SDS-PAGE using 10% polyacrylamide, and transferred to nitrocellulose membrane. ^35^S labelled BRD4 proteins were detected after exposure to film, KSHV LANAΔ33-888 [58] was detected with anti-T7•Tag antibody (Novagen), and mLANA was detected with anti-FLAG M2 antibody (Sigma).

### Generation of recombinant viruses

MuHV-4 recombinant viruses were independently generated by mutagenesis of the viral genome cloned as a bacterial artificial chromosome (BAC) [59]. The point mutations were introduced by PCR on the mLANA gene cloned into pCMV-Myc. pCMV-Myc-mLANA was digested with HindIII and PciI or BstEII and PciI to isolate the fragment harbouring the desired mutations, which were inserted into the BamHI-G genomic clone. Recombinant BamHI-G mutant fragments were subcloned into the BamHI site of a pST76K-SR shuttle plasmid and transformed into an *Escherichia coli* strain (DH10B) containing the wild-type MuHV-4 BAC (pHA3). Following a multistep selection procedure, recombinant BAC clones were identified by DNA sequencing. To generate revertant viruses the wild type BamHI-G pST76K-SR shuttle plasmid was transformed into DH10B cells containing each of the mutant BAC genomes. The integrity of each BAC was confirmed by restriction digestion with BamHI and EcoRI. All viruses were reconstituted by transfection of BAC DNA into BHK-21 cells using X-tremeGENE HP (Roche). The *loxP*-flanked BAC cassette was removed by viral passage through NIH Cre 3T3 cells and limiting dilution to obtain GFP-negative viruses.

### Analysis of recombinant viruses

We performed five independent animal infections, which were analyzed by five complementary experiments; *ex vivo* explant co-culture assays to measure virus latency in total splenocytes, flow cytometry coupled to limiting dilution and real-time PCR to quantify the frequency of viral DNA-positive in total splenocytes or to quantify GC B cells, and *in situ* hybridisation to identify virally infected cells within the spleen. *In situ* hybridisation for transcripts corresponding to the MHV-68-encoded miRNAs permits the detection of latently infected cells in splenic sections. Quantification of viral infection in GC B cells is highly relevant since mLANA is selectively expressed in proliferating GC B cells [25]. Revertant viruses were analysed at 14 days post-infection by *ex vivo* explant co-culture assay by three independent animal infections. C57BL/6 mice (Charles River, Spain) with 6–8 weeks of age were intranasally inoculated with 10^4^ p.f.u. in 20 µl of PBS under isofluorane anaesthesia. At 3, 7, 14, 21 and 50 days post-infection, lungs or spleens were removed and processed for subsequent analysis. Infectious virus titers in freeze-thawed lung homogenates were determined by suspension assay using BHK-21 cells. Latent viruses were examined using explant co-cultures of single-cell suspension splenocytes with BHK-21 cells. Plates were incubated for 4 (suspension assay) or 5 days (co-culture assay), fixed with 10% formal saline and counterstained with toluidine blue. Viral plaques were counted with a plate microscope. Frequencies of virus-genome-positive cells were determined by limiting dilution combined with real-time PCR, as described earlier [25]. Total splenocytes suspensions were prepared from pools of five spleens. GC B-cell (CD19^+^CD95^hi^GL7^hi^) populations were purified from pools of five spleens using a BD FACSAria Flow Cytometer (BD Biosciences). The purity of sorted populations was always >95%, as analysed by flow cytometry. Real-time PCR reactions were performed as reported [60]. To determine multistep growth curves, BHK-21 cells were infected with MuHV-4 recombinant viruses, at a low multiplicity of infection (0.01 p.f.u. per cell). After 1 hour of virus adsorption, cells were washed in PBS and at the indicated times post-infection virus titers were determined by plaque assay. p values were determined using ordinary one-way ANOVA (GraphPad Prism). *In situ* hybridisation was performed on formalin-fixed, paraffin-embedded splenic sessions using digoxigenin-labelled riboprobes, generated by T7 transcription of pEH1.4 [61]. *In situ* hybridisation for transcripts corresponding to the MHV-68-encoded miRNAs (encoded within pEH1.4) permits the detection of latently infected cells in splenic sessions, specifically within GC reactions.

### Accession codes

mLANA DNA AF105037; Uniprot accession numbers for mLANA O41974 and kLANA Q76SB0; The atomic coordinates and structure factors have been deposited in the Protein Data Bank under accession number PDB ID: 4blg.

## Supporting Information

Figure S1 **Construct design of mLANA.** (**A**) Schematic of mLANA truncations. (**B**) list of primer sequences used for cloning into the expression vector pET-49b(+); restriction enzyme sites are shown in bold (BamHI used in forward primers and NotI in reverse primers), extra bases to ensure the correct reading frame in red and stop codons are underlined. (**C**) Coomassie blue stained SDS-PAGE of GST-6XHis-mLANA constructs purified by Ni^2+^-sepharose beads.
(TIF)

Figure S2 **mLANA DBD homodimer analysis.** (**A**) The residues that constitute the hydrophobic core and hydrogen-bonding network of the dimer interface are coloured by amino acid group. The interactions are composed of 83 non-bonded contacts (where the interaction distance is ≤3.9 Å) with only 7 direct hydrogen bonds involved. The figure was generated by PDBSUM. (**B**) Purified mLANA DBD truncations were analysed by size exclusion chromatography. The majority of the mLANA truncations elute as a dimer, however, the elution profile of mLANA_140–314_ is consistent with a tetramer. (**C**) Biophysical characterization of mLANA protein truncations, showing the theoretical and calculated molecular weight (M_r_) of each truncation. The calculated oligomerization state and protein stability profiles are also shown. Ve – elution volume, K_av_ - partition coefficient.
(TIF)

Figure S3 **Sequence analysis of MHV-68 and KSHV LANA Binding Sites (LBS).** KSHV LBS1 and LBS2 are highlighted in blue and orange (which includes the 16 bp core) and the putative MHV-68 DNA footprints underlined in green and red [24]. Several potential mLANA LBS DNA sequences tested for energy minimization and respective result values (z-score in brackets). Energy minimization for 3D structures of mLANA-DNA models was performed using YASARA.
(TIF)

Figure S4 **Coomassie blue staining of BRD4 fusion proteins.** GST, GST-BRD4 471–594 or GST-BRD4 595–730 proteins used for binding mLANA. Similar amounts of protein used in the mLANA binding assay are shown despite staining differently with Ponceau S in Figure 4, panel E.
(TIF)

Figure S5 **Sequence comparison of the ventral and dorsal face of LANA, EBV EBNA1 and HPV6 E2 proteins.** (**A**) Table comparing the key residues of mLANA involved in DNA interactions on the ventral face showing the equivalent residues in kLANA, E2 and EBNA1 proteins. (**B**) Table comparing the key residues that contribute to the contrasting electrostatic surfaces on the dorsal face of LANA, EBNA1 and E2 proteins.
(TIF)

Figure S6 **The mLANA dimer binds cooperatively to mLBS1-2.** (**A**) The elution profile of mLANA_140–272_ dimer from size-exclusion chromatography (predicted size 31.8 kDa) in the absence of DNA. (**B**) The elution profile of mLANA_140–272_ after incubation with mLBS1, (predicted size of mLANA_140–272_ dimer-mLBS1 complex 49.4 kDa). (**C**) The elution profile of mLANA_140–272_ after incubation with mLBS1-2, (predicted size of mLANA_140–272_-mLBS1-2 complex 92.8 kDa). Samples were analysed on a Superdex 200 10/300 GL column. Peak fractions were analysed by SDS-PAGE (4–20% gradient gel) and stained for both protein (Coomassie blue) and mLBS DNA (Sybr Safe). SEC analysis confirms the binding of one mLANA_140–272_ dimer to the mLBS1 site (estimated size 51.8 kDa, V_e_ 15.8 ml) and two mLANA_140–272_ dimers to the mLBS1-2 sites (estimated size 93.6 kDa, V_e_ 13.9 ml). For panels B and C, the A_280 nm_ signal indicating protein is amplified due to the contribution of DNA in the complex, V_e_ is the sample elution volume and M represents protein standards (Supporting Information, Text S1).
(TIF)

Text S1 **Supporting data including a supporting method and supporting results.**
(DOCX)

## References

[1] Simas JP, Efstathiou S (1998) Murine gammaherpesvirus 68: a model for the study of gammaherpesvirus pathogenesis. Trends Microbiol 6: 276–282. 9717216 10.1016/s0966-842x(98)01306-7

[2] Speck SH, Ganem D (2010) Viral latency and its regulation: lessons from the gamma-herpesviruses. Cell Host Microbe 8: 100–115. 20638646 10.1016/j.chom.2010.06.014 PMC2914632

[3] Nash AA, Dutia BM, Stewart JP, Davison AJ (2001) Natural history of murine gamma-herpesvirus infection. Philos Trans R Soc Lond B Biol Sci 356: 569–579. 11313012 10.1098/rstb.2000.0779 PMC1088445

[4] Cardin RD, Brooks JW, Sarawar SR, Doherty PC (1996) Progressive loss of CD8+ T cell-mediated control of a gamma-herpesvirus in the absence of CD4+ T cells. J Exp Med 184: 863–871. 9064346 10.1084/jem.184.3.863 PMC2192775

[5] Sunil-Chandra NP, Efstathiou S, Nash AA (1992) Murine gammaherpesvirus 68 establishes a latent infection in mouse B lymphocytes in vivo. J Gen Virol 73 Pt 12: 3275–3279. 1469366 10.1099/0022-1317-73-12-3275

[6] Flano E, Kim IJ, Woodland DL, Blackman MA (2002) Gamma-herpesvirus latency is preferentially maintained in splenic germinal center and memory B cells. J Exp Med 196: 1363–1372. 12438427 10.1084/jem.20020890 PMC2193987

[7] Thorley-Lawson DA (2001) Epstein-Barr virus: exploiting the immune system. Nat Rev Immunol 1: 75–82. 11905817 10.1038/35095584

[8] Ballestas ME, Kaye KM (2001) Kaposi's sarcoma-associated herpesvirus latency-associated nuclear antigen 1 mediates episome persistence through cis-acting terminal repeat (TR) sequence and specifically binds TR DNA. J Virol 75: 3250–3258. 11238851 10.1128/JVI.75.7.3250-3258.2001 PMC114118

[9] Cotter MAII, Subramanian C, Robertson ES (2001) The Kaposi's sarcoma-associated herpesvirus latency-associated nuclear antigen binds to specific sequences at the left end of the viral genome through its carboxy-terminus. Virology 291: 241–259. 11878894 10.1006/viro.2001.1202

[10] Garber AC, Hu J, Renne R (2002) Latency-associated nuclear antigen (LANA) cooperatively binds to two sites within the terminal repeat, and both sites contribute to the ability of LANA to suppress transcription and to facilitate DNA replication. J Biol Chem 277: 27401–27411. 12015325 10.1074/jbc.M203489200

[11] Hu J, Garber AC, Renne R (2002) The latency-associated nuclear antigen of Kaposi's sarcoma-associated herpesvirus supports latent DNA replication in dividing cells. J Virol 76: 11677–11687. 12388727 10.1128/JVI.76.22.11677-11687.2002 PMC136756

[12] Komatsu T, Ballestas ME, Barbera AJ, Kelley-Clarke B, Kaye KM (2004) KSHV LANA1 binds DNA as an oligomer and residues N-terminal to the oligomerization domain are essential for DNA binding, replication, and episome persistence. Virology 319: 225–236. 14980483 10.1016/j.virol.2003.11.002

[13] Lim C, Sohn H, Lee D, Gwack Y, Choe J (2002) Functional dissection of latency-associated nuclear antigen 1 of Kaposi's sarcoma-associated herpesvirus involved in latent DNA replication and transcription of terminal repeats of the viral genome. J Virol 76: 10320–10331. 12239308 10.1128/JVI.76.20.10320-10331.2002 PMC136563

[14] Schwam DR, Luciano RL, Mahajan SS, Wong L, Wilson AC (2000) Carboxy terminus of human herpesvirus 8 latency-associated nuclear antigen mediates dimerization, transcriptional repression, and targeting to nuclear bodies. J Virol 74: 8532–8540. 10954554 10.1128/jvi.74.18.8532-8540.2000 PMC116365

[15] Barbera AJ, Chodaparambil JV, Kelley-Clarke B, Joukov V, Walter JC, et al (2006) The nucleosomal surface as a docking station for Kaposi's sarcoma herpesvirus LANA. Science 311: 856–861. 16469929 10.1126/science.1120541

[16] Ballestas ME, Chatis PA, Kaye KM (1999) Efficient persistence of extrachromosomal KSHV DNA mediated by latency-associated nuclear antigen. Science 284: 641–644. 10213686 10.1126/science.284.5414.641

[17] Cotter MAII, Robertson ES (1999) The latency-associated nuclear antigen tethers the Kaposi's sarcoma-associated herpesvirus genome to host chromosomes in body cavity-based lymphoma cells. Virology 264: 254–264. 10562490 10.1006/viro.1999.9999

[18] Hung SC, Kang MS, Kieff E (2001) Maintenance of Epstein-Barr virus (EBV) oriP-based episomes requires EBV-encoded nuclear antigen-1 chromosome-binding domains, which can be replaced by high-mobility group-I or histone H1. Proc Natl Acad Sci U S A 98: 1865–1870. 11172042 10.1073/pnas.031584698 PMC29348

[19] Ilves I, Kivi S, Ustav M (1999) Long-term episomal maintenance of bovine papillomavirus type 1 plasmids is determined by attachment to host chromosomes, which Is mediated by the viral E2 protein and its binding sites. J Virol 73: 4404–4412. 10196338 10.1128/jvi.73.5.4404-4412.1999 PMC104221

[20] Lehman CW, Botchan MR (1998) Segregation of viral plasmids depends on tethering to chromosomes and is regulated by phosphorylation. Proc Natl Acad Sci U S A 95: 4338–4343. 9539738 10.1073/pnas.95.8.4338 PMC22490

[21] Yates JL, Warren N, Sugden B (1985) Stable replication of plasmids derived from Epstein-Barr virus in various mammalian cells. Nature 313: 812–815. 2983224 10.1038/313812a0

[22] Bastien N, McBride AA (2000) Interaction of the papillomavirus E2 protein with mitotic chromosomes. Virology 270: 124–134. 10772985 10.1006/viro.2000.0265

[23] Habison AC, Beauchemin C, Simas JP, Usherwood EJ, Kaye KM (2012) Murine Gammaherpesvirus 68 LANA Acts on Terminal Repeat DNA To Mediate Episome Persistence. J Virol 86: 11863–11876. 22915819 10.1128/JVI.01656-12 PMC3486315

[24] Paden CR, Forrest JC, Tibbetts SA, Speck SH (2012) Unbiased Mutagenesis of MHV68 LANA Reveals a DNA-Binding Domain Required for LANA Function In Vitro and In Vivo. PLoS Pathog 8: e1002906. 22969427 10.1371/journal.ppat.1002906 PMC3435236

[25] Marques S, Efstathiou S, Smith KG, Haury M, Simas JP (2003) Selective gene expression of latent murine gammaherpesvirus 68 in B lymphocytes. J Virol 77: 7308–7318. 12805429 10.1128/JVI.77.13.7308-7318.2003 PMC164786

[26] Fowler P, Marques S, Simas JP, Efstathiou S (2003) ORF73 of murine herpesvirus-68 is critical for the establishment and maintenance of latency. J Gen Virol 84: 3405–3416. 14645921 10.1099/vir.0.19594-0

[27] Moorman NJ, Willer DO, Speck SH (2003) The gammaherpesvirus 68 latency-associated nuclear antigen homolog is critical for the establishment of splenic latency. J Virol 77: 10295–10303. 12970414 10.1128/JVI.77.19.10295-10303.2003 PMC228443

[28] An FQ, Compitello N, Horwitz E, Sramkoski M, Knudsen ES, et al (2005) The latency-associated nuclear antigen of Kaposi's sarcoma-associated herpesvirus modulates cellular gene expression and protects lymphoid cells from p16 INK4A-induced cell cycle arrest. J Biol Chem 280: 3862–3874. 15525642 10.1074/jbc.M407435200

[29] Shamay M, Krithivas A, Zhang J, Hayward SD (2006) Recruitment of the de novo DNA methyltransferase Dnmt3a by Kaposi's sarcoma-associated herpesvirus LANA. Proc Natl Acad Sci U S A 103: 14554–14559. 16983096 10.1073/pnas.0604469103 PMC1599998

[30] Cai QL, Knight JS, Verma SC, Zald P, Robertson ES (2006) EC5S ubiquitin complex is recruited by KSHV latent antigen LANA for degradation of the VHL and p53 tumor suppressors. PLoS Pathog 2: e116. 17069461 10.1371/journal.ppat.0020116 PMC1626105

[31] Rodrigues L, Filipe J, Seldon MP, Fonseca L, Anrather J, et al (2009) Termination of NF-kappaB activity through a gammaherpesvirus protein that assembles an EC5S ubiquitin-ligase. EMBO J 28: 1283–1295. 19322197 10.1038/emboj.2009.74 PMC2664658

[32] Li X, Liang D, Lin X, Robertson ES, Lan K (2011) Kaposi's sarcoma-associated herpesvirus-encoded latency-associated nuclear antigen reduces interleukin-8 expression in endothelial cells and impairs neutrophil chemotaxis by degrading nuclear p65. J Virol 85: 8606–8615. 21697472 10.1128/JVI.00733-11 PMC3165807

[33] Ottinger M, Christalla T, Nathan K, Brinkmann MM, Viejo-Borbolla A, et al (2006) Kaposi's sarcoma-associated herpesvirus LANA-1 interacts with the short variant of BRD4 and releases cells from a BRD4- and BRD2/RING3-induced G1 cell cycle arrest. J Virol 80: 10772–10786. 16928766 10.1128/JVI.00804-06 PMC1641788

[34] Ottinger M, Pliquet D, Christalla T, Frank R, Stewart JP, et al (2009) The interaction of the gammaherpesvirus 68 orf73 protein with cellular BET proteins affects the activation of cell cycle promoters. J Virol 83: 4423–4434. 19244327 10.1128/JVI.02274-08 PMC2668493

[35] Grundhoff A, Ganem D (2003) The latency-associated nuclear antigen of Kaposi's sarcoma-associated herpesvirus permits replication of terminal repeat-containing plasmids. J Virol 77: 2779–2783. 12552022 10.1128/JVI.77.4.2779-2783.2003 PMC141125

[36] Bochkarev A, Barwell JA, Pfuetzner RA, Furey W Jr, Edwards AM, et al (1995) Crystal structure of the DNA-binding domain of the Epstein-Barr virus origin-binding protein EBNA 1. Cell 83: 39–46. 7553871 10.1016/0092-8674(95)90232-5

[37] Dell G, Wilkinson KW, Tranter R, Parish J, Leo Brady R, et al (2003) Comparison of the structure and DNA-binding properties of the E2 proteins from an oncogenic and a non-oncogenic human papillomavirus. J Mol Biol 334: 979–991. 14643661 10.1016/j.jmb.2003.10.009

[38] Reisman D, Yates J, Sugden B (1985) A putative origin of replication of plasmids derived from Epstein-Barr virus is composed of two cis-acting components. Mol Cell Biol 5: 1822–1832. 3018528 10.1128/mcb.5.8.1822-1832.1985 PMC366897

[39] Han SJ, Hu J, Pierce B, Weng Z, Renne R (2010) Mutational analysis of the latency-associated nuclear antigen DNA-binding domain of Kaposi's sarcoma-associated herpesvirus reveals structural conservation among gammaherpesvirus origin-binding proteins. J Gen Virol 91: 2203–2215. 20484563 10.1099/vir.0.020958-0 PMC3066550

[40] Kelley LA, Sternberg MJ (2009) Protein structure prediction on the Web: a case study using the Phyre server. Nat Protoc 4: 363–371. 19247286 10.1038/nprot.2009.2

[41] You J, Srinivasan V, Denis GV, Harrington WJ Jr, Ballestas ME, et al (2006) Kaposi's sarcoma-associated herpesvirus latency-associated nuclear antigen interacts with bromodomain protein Brd4 on host mitotic chromosomes. J Virol 80: 8909–8919. 16940503 10.1128/JVI.00502-06 PMC1563901

[42] Kelley-Clarke B, De Leon-Vazquez E, Slain K, Barbera AJ, Kaye KM (2009) Role of Kaposi's sarcoma-associated herpesvirus C-terminal LANA chromosome binding in episome persistence. J Virol 83: 4326–4337. 19225000 10.1128/JVI.02395-08 PMC2668501

[43] Kelley-Clarke B, Ballestas ME, Srinivasan V, Barbera AJ, Komatsu T, et al (2007) Determination of Kaposi's sarcoma-associated herpesvirus C-terminal latency-associated nuclear antigen residues mediating chromosome association and DNA binding. J Virol 81: 4348–4356. 17287261 10.1128/JVI.01289-06 PMC1866165

[44] Stebbins CE, Kaelin WG Jr, Pavletich NP (1999) Structure of the VHL-ElonginC-ElonginB complex: implications for VHL tumor suppressor function. Science 284: 455–461. 10205047 10.1126/science.284.5413.455

[45] Stanley BJ, Ehrlich ES, Short L, Yu Y, Xiao Z, et al (2008) Structural insight into the human immunodeficiency virus Vif SOCS box and its role in human E3 ubiquitin ligase assembly. J Virol 82: 8656–8663. 18562529 10.1128/JVI.00767-08 PMC2519636

[46] Lin A, Wang S, Nguyen T, Shire K, Frappier L (2008) The EBNA1 protein of Epstein-Barr virus functionally interacts with Brd4. J Virol 82: 12009–12019. 18922874 10.1128/JVI.01680-08 PMC2593323

[47] Baxter MK, McPhillips MG, Ozato K, McBride AA (2005) The mitotic chromosome binding activity of the papillomavirus E2 protein correlates with interaction with the cellular chromosomal protein, Brd4. J Virol 79: 4806–4818. 15795266 10.1128/JVI.79.8.4806-4818.2005 PMC1069523

[48] Kabsch W (2010) Xds. Acta Crystallogr D Biol Crystallogr 66: 125–132. 20124692 10.1107/S0907444909047337 PMC2815665

[49] Evans P (2006) Scaling and assessment of data quality. Acta Crystallogr D Biol Crystallogr 62: 72–82. 16369096 10.1107/S0907444905036693

[50] McCoy AJ, Grosse-Kunstleve RW, Adams PD, Winn MD, Storoni LC, et al (2007) Phaser crystallographic software. J Appl Crystallogr 40: 658–674. 19461840 10.1107/S0021889807021206 PMC2483472

[51] Adams PD, Afonine PV, Bunkoczi G, Chen VB, Davis IW, et al (2010) PHENIX: a comprehensive Python-based system for macromolecular structure solution. Acta Crystallogr D Biol Crystallogr 66: 213–221. 20124702 10.1107/S0907444909052925 PMC2815670

[52] Emsley P, Cowtan K (2004) Coot: model-building tools for molecular graphics. Acta Crystallogr D Biol Crystallogr 60: 2126–2132. 15572765 10.1107/S0907444904019158

[53] Chen VB, Arendall WB (2010) MolProbity: all-atom structure validation for macromolecular crystallography. Acta Crystallogr D Biol Crystallogr 66: 12–21. 20057044 10.1107/S0907444909042073 PMC2803126

[54] Krissinel E, Henrick K (2007) Inference of macromolecular assemblies from crystalline state. J Mol Biol 372: 774–797. 17681537 10.1016/j.jmb.2007.05.022

[55] Krieger E, Joo K, Lee J, Raman S, Thompson J, et al (2009) Improving physical realism, stereochemistry, and side-chain accuracy in homology modeling: Four approaches that performed well in CASP8. Proteins 77 Suppl 9: 114–122. 19768677 10.1002/prot.22570 PMC2922016

[56] Schrödinger, LLC (2010) The PyMOL Molecular Graphics System, Version 1.5.0.3.

[57] McNicholas S, Potterton E, Wilson KS, Noble ME (2011) Presenting your structures: the CCP4mg molecular-graphics software. Acta Crystallogr D Biol Crystallogr 67: 386–394. 21460457 10.1107/S0907444911007281 PMC3069754

[58] De Leon Vazquez E, Kaye KM (2011) The internal Kaposi's sarcoma-associated herpesvirus LANA regions exert a critical role on episome persistence. J Virol 85: 7622–7633. 21593163 10.1128/JVI.00304-11 PMC3147901

[59] Adler H, Messerle M, Wagner M, Koszinowski UH (2000) Cloning and mutagenesis of the murine gammaherpesvirus 68 genome as an infectious bacterial artificial chromosome. J Virol 74: 6964–6974. 10888635 10.1128/jvi.74.15.6964-6974.2000 PMC112213

[60] Pires de Miranda M, Alenquer M, Marques S, Rodrigues L, Lopes F, et al (2008) The Gammaherpesvirus m2 protein manipulates the Fyn/Vav pathway through a multidocking mechanism of assembly. PLoS One 3: e1654. 18301737 10.1371/journal.pone.0001654 PMC2244710

[61] Simas JP, Bowden RJ, Paige V, Efstathiou S (1998) Four tRNA-like sequences and a serpin homologue encoded by murine gammaherpesvirus 68 are dispensable for lytic replication in vitro and latency in vivo. J Gen Virol 79 Pt 1: 149–153. 9460936 10.1099/0022-1317-79-1-149

[62] Wu SY, Chiang CM (2007) The double bromodomain-containing chromatin adaptor Brd4 and transcriptional regulation. J Biol Chem 282: 13141–13145. 17329240 10.1074/jbc.R700001200

[63] Belkina AC, Denis GV (2012) BET domain co-regulators in obesity, inflammation and cancer. Nat Rev Cancer 12: 465–477. 22722403 10.1038/nrc3256 PMC3934568

