# Supplementary figures and images for: Crystal Structure of the Gamma-2 Herpesvirus LANA DNA Binding Domain Identifies Charged Surface Residues Which Impact Viral Latency
Source: PLoS Pathog. 2013 Oct 17;9(10):e1003673. doi: 10.1371/journal.ppat.1003673 (PMC3798461; doi:10.1371/journal.ppat.1003673)

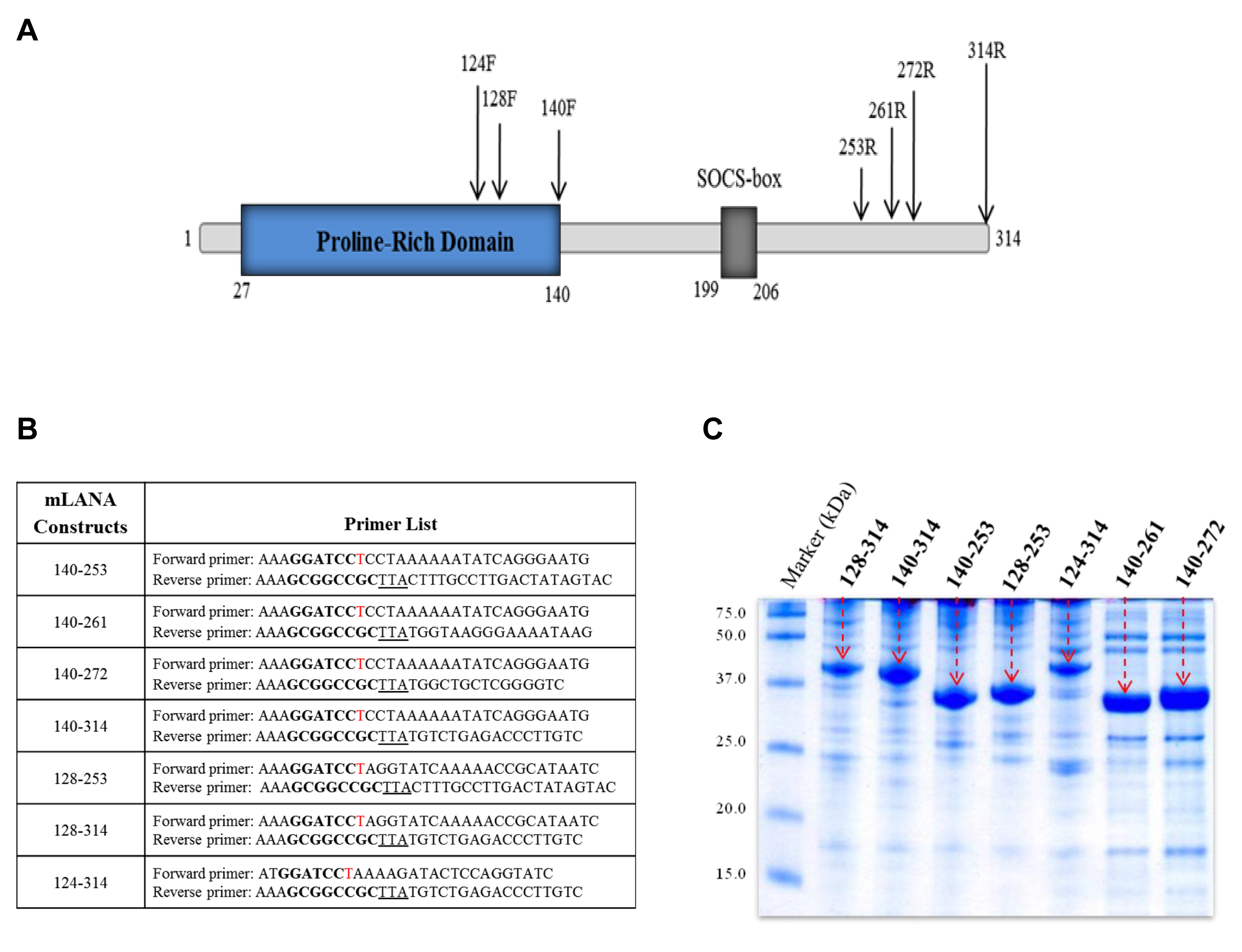

Supplement: Figure S1 — Construct design of mLANA. (A) Schematic of mLANA truncations. (B) list of primer sequences used for cloning into the expression vector pET-49b(+); restriction enzyme sites are shown in bold (BamHI used in forward primers and NotI in reverse primers), extra bases to ensure the correct reading frame in red and stop codons are underlined. (C) Coomassie blue stained SDS-PAGE of GST-6XHis-mLANA constructs purified by Ni2+-sepharose beads. (TIF) [file ppat.1003673.s001.tif]

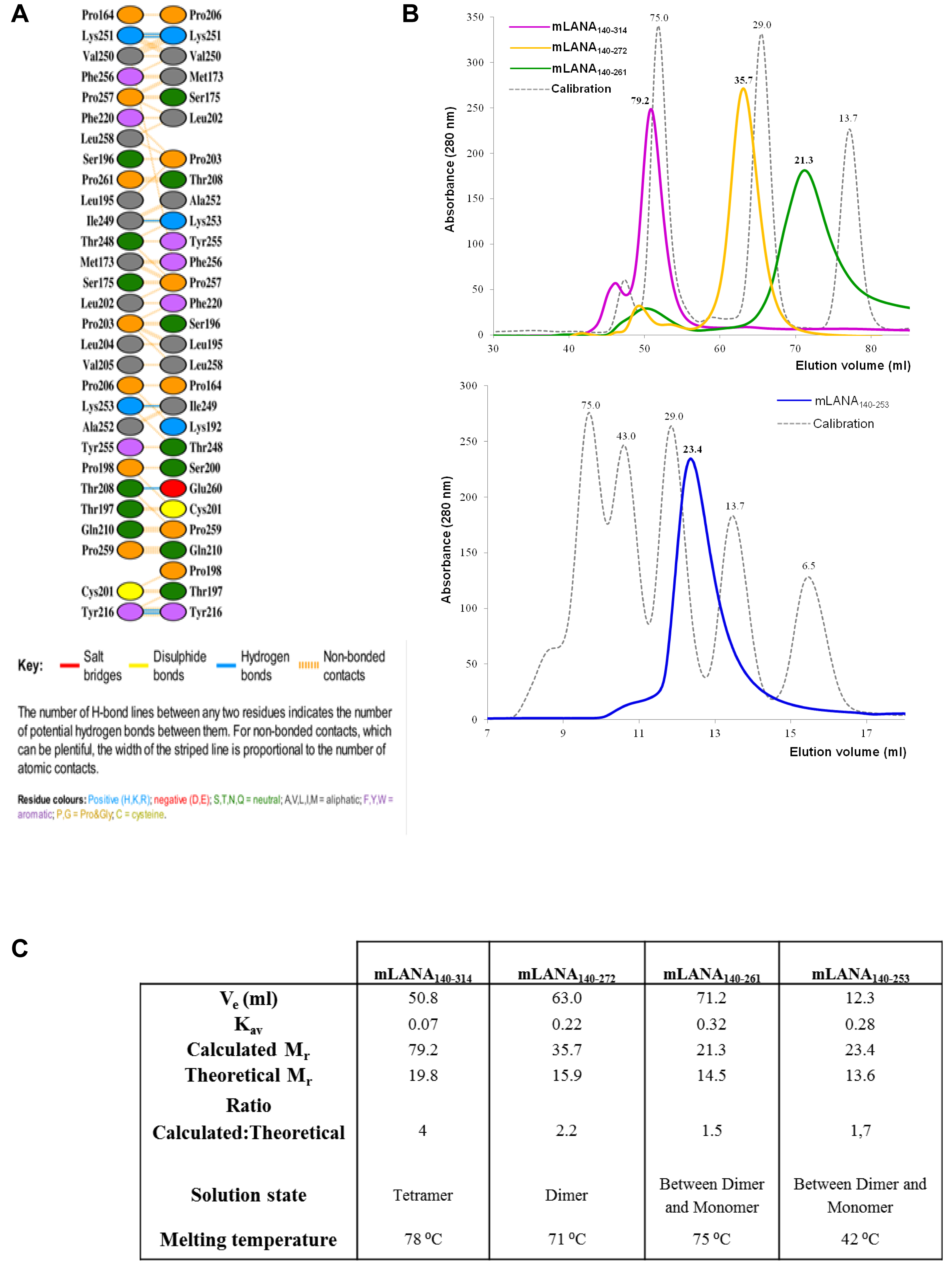

Supplement: Figure S2 — mLANA DBD homodimer analysis. (A) The residues that constitute the hydrophobic core and hydrogen-bonding network of the dimer interface are coloured by amino acid group. The interactions are composed of 83 non-bonded contacts (where the interaction distance is ≤3.9 Å) with only 7 direct hydrogen bonds involved. The figure was generated by PDBSUM. (B) Purified mLANA DBD truncations were analysed by size exclusion chromatography. The majority of the mLANA truncations elute as a dimer, however, the elution profile of mLANA140–314 is consistent with a tetramer. (C) Biophysical characterization of mLANA protein truncations, showing the theoretical and calculated molecular weight (Mr) of each truncation. The calculated oligomerization state and protein stability profiles are also shown. Ve – elution volume, Kav - partition coefficient. (TIF) [file ppat.1003673.s002.tif]

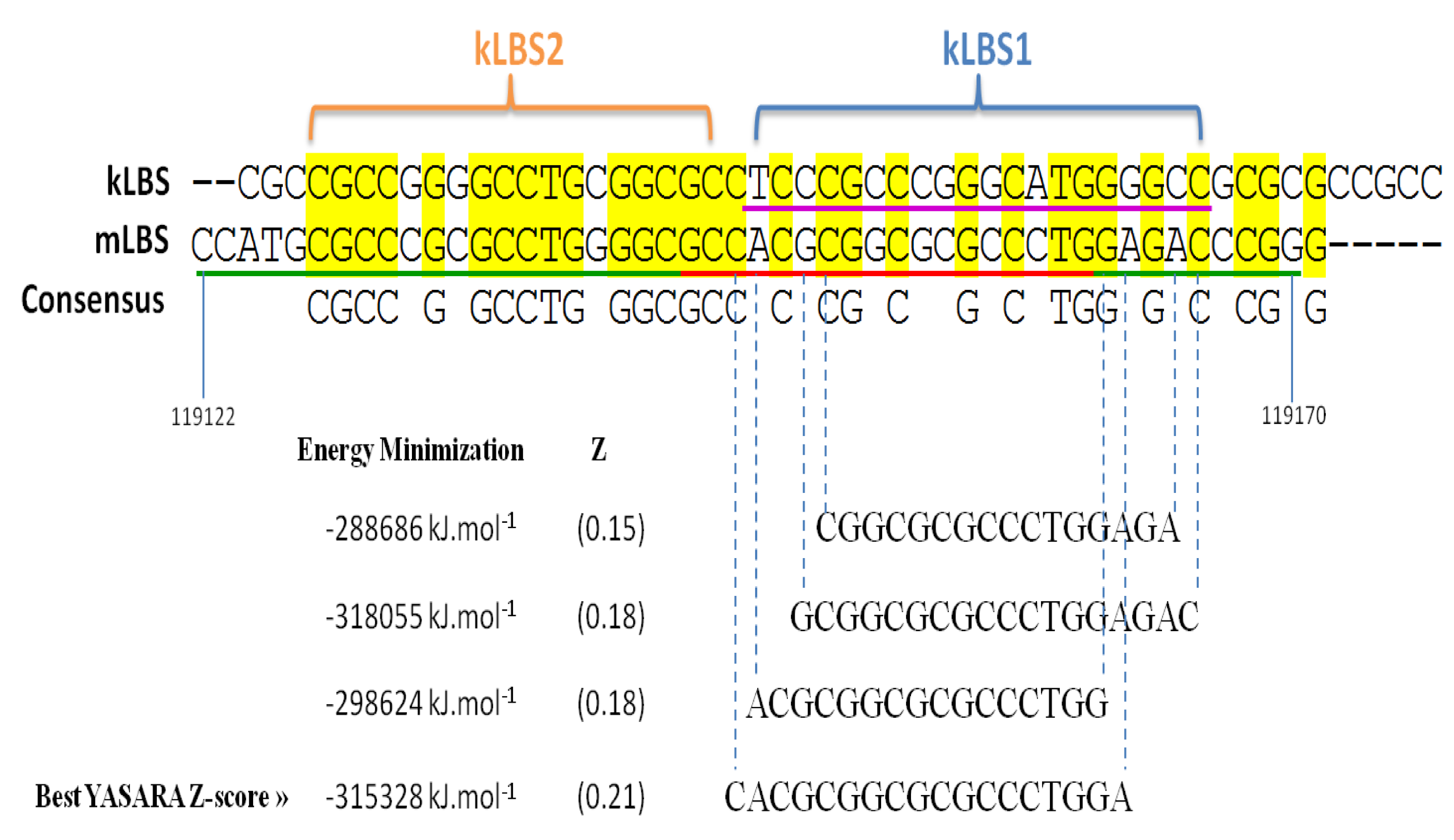

Supplement: Figure S3 — Sequence analysis of MHV-68 and KSHV LANA Binding Sites (LBS). KSHV LBS1 and LBS2 are highlighted in blue and orange (which includes the 16 bp core) and the putative MHV-68 DNA footprints underlined in green and red [24]. Several potential mLANA LBS DNA sequences tested for energy minimization and respective result values (z-score in brackets). Energy minimization for 3D structures of mLANA-DNA models was performed using YASARA. (TIF) [file ppat.1003673.s003.tif]

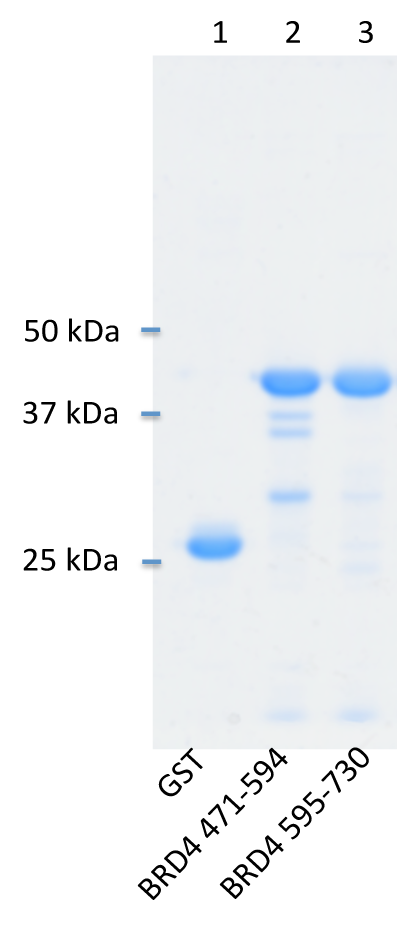

Supplement: Figure S4 — Coomassie blue staining of BRD4 fusion proteins. GST, GST-BRD4 471–594 or GST-BRD4 595–730 proteins used for binding mLANA. Similar amounts of protein used in the mLANA binding assay are shown despite staining differently with Ponceau S in Figure 4, panel E. (TIF) [file ppat.1003673.s004.tif]

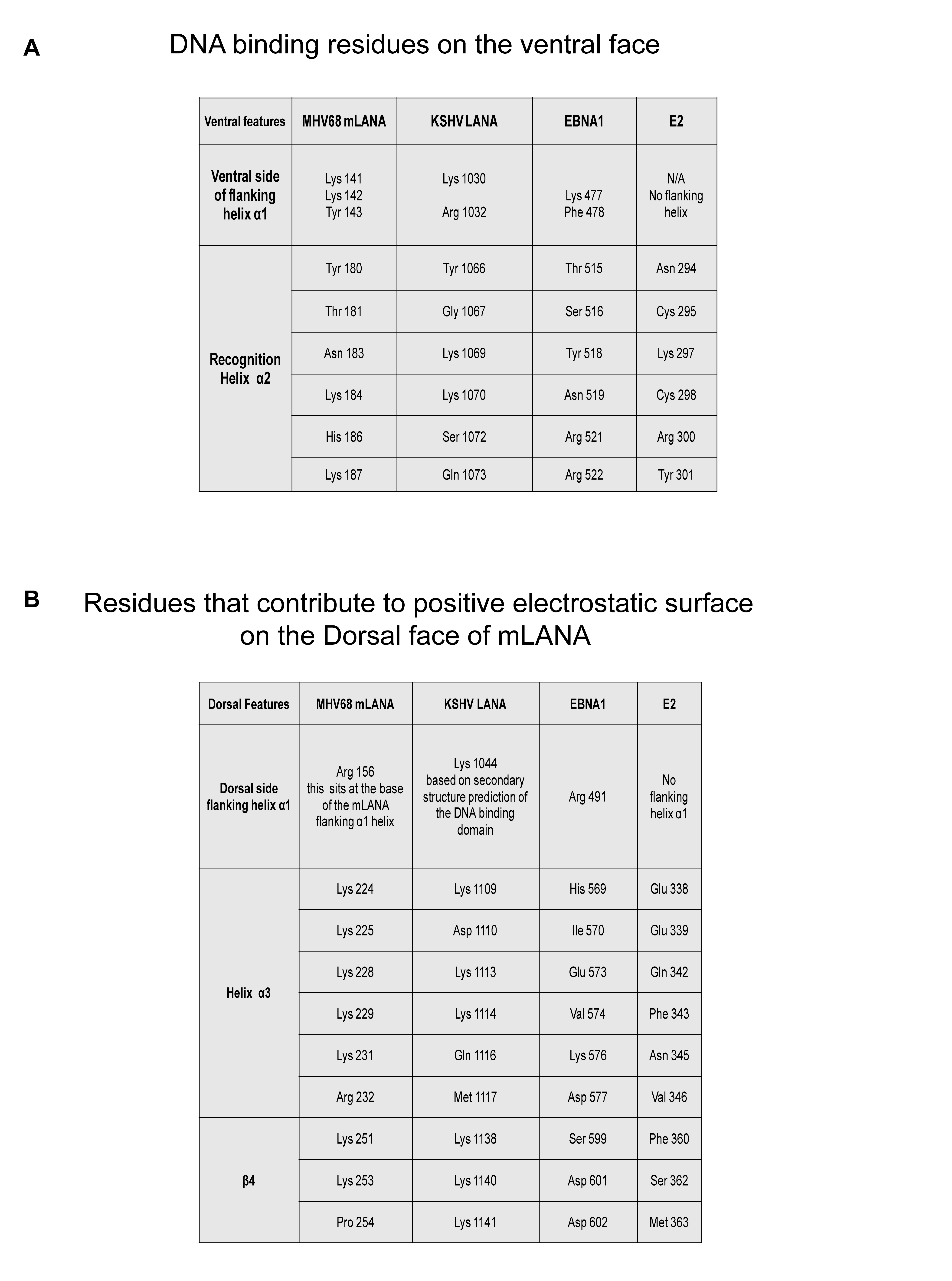

Supplement: Figure S5 — Sequence comparison of the ventral and dorsal face of LANA, EBV EBNA1 and HPV6 E2 proteins. (A) Table comparing the key residues of mLANA involved in DNA interactions on the ventral face showing the equivalent residues in kLANA, E2 and EBNA1 proteins. (B) Table comparing the key residues that contribute to the contrasting electrostatic surfaces on the dorsal face of LANA, EBNA1 and E2 proteins. (TIF) [file ppat.1003673.s005.tif]

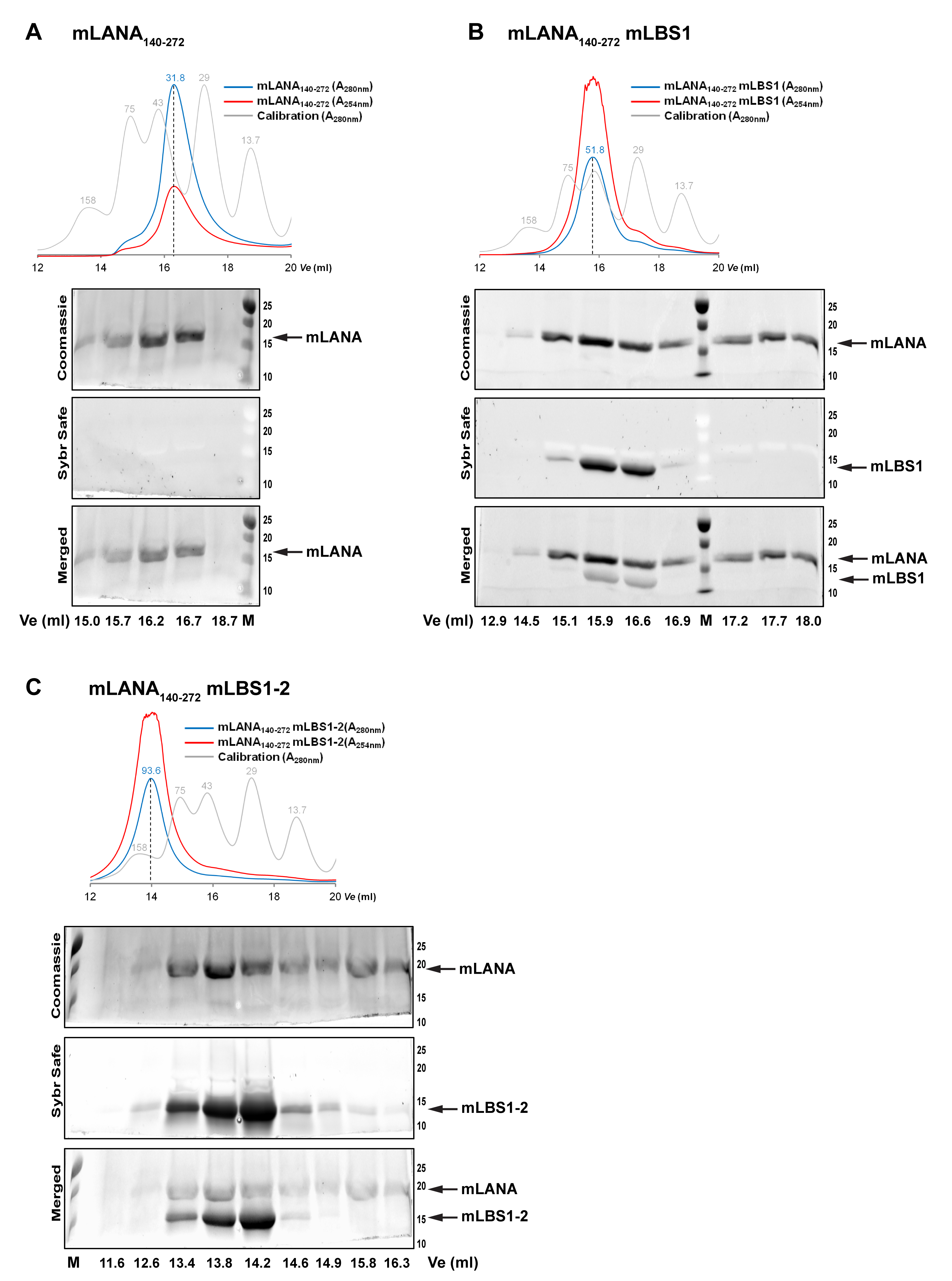

Supplement: Figure S6 — The mLANA dimer binds cooperatively to mLBS1-2. (A) The elution profile of mLANA140–272 dimer from size-exclusion chromatography (predicted size 31.8 kDa) in the absence of DNA. (B) The elution profile of mLANA140–272 after incubation with mLBS1, (predicted size of mLANA140–272 dimer-mLBS1 complex 49.4 kDa). (C) The elution profile of mLANA140–272 after incubation with mLBS1-2, (predicted size of mLANA140–272-mLBS1-2 complex 92.8 kDa). Samples were analysed on a Superdex 200 10/300 GL column. Peak fractions were analysed by SDS-PAGE (4–20% gradient gel) and stained for both protein (Coomassie blue) and mLBS DNA (Sybr Safe). SEC analysis confirms the binding of one mLANA140–272 dimer to the mLBS1 site (estimated size 51.8 kDa, Ve 15.8 ml) and two mLANA140–272 dimers to the mLBS1-2 sites (estimated size 93.6 kDa, Ve 13.9 ml). For panels B and C, the A280 nm signal indicating protein is amplified due to the contribution of DNA in the complex, Ve is the sample elution volume and M represents protein standards (Supporting Information, Text S1). (TIF) [file ppat.1003673.s006.tif]
