# Supplementary material for: Crystal Structure of the Gamma-2 Herpesvirus LANA DNA Binding Domain Identifies Charged Surface Residues Which Impact Viral Latency
Source: PLoS Pathog. 2013 Oct 17;9(10):e1003673. doi: 10.1371/journal.ppat.1003673 (PMC3798461; doi:10.1371/journal.ppat.1003673)
Supplement: Text S1 — Supporting data including a supporting method and supporting results. (DOCX) [file ppat.1003673.s007.docx]

## Supporting Methods

**Cooperative binding analysis of C-terminal mLANA**

We used size exclusion chromatography (SEC) to examine cooperative binding of mLANA DBD to mLBS1 and mLBS1-2 DNA. The samples were analysed on a S200 10/300 GL column (24 ml bed volume) equilibrated with buffer containing 20 mM Na/K phosphate pH 7.0, 300mM NaCl. mLANA_140-272_ was incubated alone, with mLBS1 or with mLBS1-2 (see Figure 3A) overnight on ice before being applied to the column. The elution was analyzed by monitoring UV absorbance at 280

nm and 254

nm using an ÄKTA Explorer (GE Healthcare) FPLC system for protein and DNA respectively. The column was calibrated using gel-filtration protein standards (Sigma). The elution volumes (*Ve*) for mLANA_140-272_ and mLANA_140-272_ - DNA complexes were used to determine the molecular weights of the complexes using the calibration curve. Fractions were analysed by SDS-PAGE (5-20% gradient gel) and stained with both Coomassie blue and Sybr Safe (Invitrogen) for protein and DNA respectively. Gels were scanned with a Fuji FLA-5100 instrument at wavelengths 473 nm (LPB filter for DNA fluorescence), 525 nm (LPFR filter for merged) and 635 nm (LPFR filter for protein).

## Supporting Results

**Dimer interface of C-terminal mLANA**

Analysis of the interactions within the dimer interface was performed using the PISA server. Monomer A and B form a dimer stabilized by an interface with a surface area per monomer (~1438 Å^2^) that is approximately 19% of that of the total surface of a single monomer (~7,500 Å^2^). Residues on both β2 and β4 of monomer A interact with residues on the equivalent strand of the adjacent monomer B to form the β-barrel core. Thirty amino acids from each subunit participate in around 83 non-bonded contacts (where the interaction distance is ≤3.9Å) and only 7 direct hydrogen bonds (Figure S2A). The β-barrel in the mLANA has incomplete closure as there are no main-chain hydrogen bonds present on the ventral side between strands β2 of each monomer to close the β-barrel and only main-chain hydrogen bonds on the dorsal side of the dimer between strands β4 of each monomer (residues Ile-249, 2 hydrogen bonds from Lys-251 and Lys-253). This can be attributed to a β-bulge which interrupts β2 strand (Figure 2B). Of note, this bulge feature is observed in the structure of both papillomavirus E2 and EBNA1 proteins, which share structural homology [[1](#_ENREF_1),[2](#_ENREF_2)], and may be in part a functional characteristic since it lies on the putative DNA binding face. Size exclusion analysis shows that mLANA_140-272_ is also a dimer in solution (Figure S2B and C). DSF analysis of the mLANA dimer revealed high thermal stability (T_m_ ~80°C, Figure 1D).

**Dimer-dimer interaction**

The presumed dimer-dimer interaction, together with the symmetry-related equivalent, would result in the formation of a helix bundle that stabilizes the dimer-dimer interaction on DNA. Size exclusion analysis of mLANA_140-314_ shows that mLANA can form a tetramer in solution (Figure S2). PISA analysis of the tetramer interface shows that the two dimers form a tetramer stabilized by an interface with a surface area (~535 Å^2^) per monomer that is approximately 12% of that of the total surface of a single subunit (~7,500 Å^2^). 15 amino acids from each dimer participate in around 11 non-bonded contacts and 6 direct hydrogen bonds.

**Structural similarities between C-terminal mLANA and EBNA1**

mLANA and EBNA1 share a common quaternary structure (average r.m.s. deviation 1.37Å when mLANA and EBNA1 dimers are compared based on 164 equivalent Cα atoms). They both share the same mode of dimerization in which the β-barrel forms the nucleus of the protein dimer. A noticeable feature of the hydrophobic interior is tyrosine Tyr-216 located on strand β3. The side-chain of this residue spans across the top of the β-barrel and hydrogen bonds (2.7 Å) with Tyr-216 of the other monomer and forms two of only seven hydrogen bonds present in the dimer interface (Figure 2B and S2). This is identical to the interaction observed in EBNA1 involving Tyr-561 (2.8 Å) and its dimer equivalent.

A structure-based sequence alignment of the DNA-binding domains of mLANA and EBNA1 identifies common sequence motifs in helix α2 and in strand β2 (Figure 2A and S5A). In both mLANA and EBNA1, there is a bulge in the β2 strand as a result of the insertion of a proline (Pro-198) in the strand (Figure 2B). This proline amide breaks the inter-strand hydrogen bond pattern between the backbone atoms and results in a side-chain (Thr-197-OH; β2) and main-chain (Leu-218-NH; β3) inter-strand hydrogen bond which prevents β-barrel closure. Interestingly, the bulge in β strand lies on the proposed DNA binding face of mLANA and may have a role in DNA interactions.

**mLANA SOCS loop**

A requirement of Elongin C binding with HIV Vif is the insertion of a leucine residue in the hydrophobic pocket of Elongin C, this region is disordered in Vif prior to Elongin C binding [[3](#_ENREF_3)]. Leu-204 of mLANA is a possible candidate for Elongin C interaction as it is ideally positioned for a hydrophobic interaction. This loop is a unique structural feature of mLANA as the equivalent β2-β3 loop in EBNA1 is larger by 8 residues and contains a proline rich sequence (PGPGP) that is absent from mLANA (Figure 2A and 2C). The longer EBNA1 loop also has a single twist over strand β2 back to β3, not observed in the mLANA loop (Figure 2C).

**The ventral mLANA DNA binding face**

The phosphate binding pattern observed in the structure allows identification and comparison of key residues likely to be involved in DNA interaction along the ventral face (Figure S5A). The recognition helix α2 interacts with three phosphate ions, which hydrogen bond with the side-chain residues His-178, Thr-181, Asn-183, Lys-184 and Lys-187 (Figure 2F). The five ventral facing phosphate ions found in the structure mimic the phosphate backbone of bound DNA with the positive electrostatic surface localised to the recognition helix and suggests that the central β-barrel does not interact directly with the DNA (Figure 2D and E).

Modelling the mLANA-DNA complex based on the EBNA1 structure (Fig. 2G) shed light on the likely structural relationship of mLANA with TR DNA. Molecular docking of the TR DNA was based on the nucleotide substitution of the EBNA DNA with the resulting mLANA-DNA models evaluated with YASARA. The mLANA DNA-binding domain accommodates the modified EBNA1 DNA convincingly and like EBNA1, the β-barrel core is oriented to contact the inner portion of the LANA DNA binding site with key base specific interactions implemented by the α1 flanking helices. The EBNA1 DNA sequence was mutated in Coot to the corresponding mLANA DNA sequences identified recently (Figure S3). The model shows that helix α1 is oriented suitably to contact the outer two or three bases of the LANA DNA-binding site, with the N-terminus of the helix composed of positively charged residues in very close proximity to the bases 16-17, while the recognition helix α2 fits snugly into the DNA major groove. Both the flanking helix (Lys-142) and recognition helix (Asn-183, His-186 and Lys-187) are well positioned to hydrogen bond with the DNA (Figure 2G). However, the proposed recognition helix α2 of mLANA is four residues shorter (one helical turn) compared to the EBNA1 recognition helix and this may influence the specificity towards its cognate DNA.

**SPR and ITC K_D_ values**

The ITC calculated dissociation constant was 66.0 ± 12 nM with an n value of 0.27 ± 0.03 sites mLANA_124-314_ dimer per mLBS1. The binding event is enthalpy driven, indicative of H-bonding coupling to a conformational change upon DNA binding as seen from the calculated thermodynamics parameters (ΔH^0^: -103.9 ± 3.4 kJ/mol, -ΔTS^0^: 63.0 ±3.4 kJ/mol, ΔG^0^: -41.0 ± 0.5 kJ/mol). The SPR K_D_ of 169 nM was substantially higher than the ITC K_D_ of 66 nM and the discrepancy was primarily due to the inaccessibility of more than half of the mLANA_124-314_ for DNA binding. This finding may be due to mLANA_124-314_ forming higher order oligomers (most likely tetramers), upon injection, despite the use of mLANA immediately taken from the dimer peak after size exclusion chromatography purification. Unlike ITC, the SPR dissociation constant is mainly determined by analyte concentration (i.e., mLANA); therefore the calculated value is higher.

**Phenotypes of recombinant virus with loss of DNA binding**

vmLANA_H186D/K187E_ replicated to higher levels than WT for the first 48 hours, followed by a growth plateau, while WT virus reached a slightly higher growth plateau at 96 hours (Figure 5B). This result differs from previous observations that reported that ORF73 null virus or virus carrying point mutations disrupting mLANA binding to cognate DNA [[4](#_ENREF_4)] replicated with lower titres upon low multiplicity of infection (MOI of 0.001PFU) of fibroblasts. It is possible that the difference between these studies and our study reflect differences in the MOI or the cell line utilized. Here we utilized an MOI of 0.01PFU to infect baby hamster kidney cells. Interestingly, lytic replication was reduced in lungs when compared to wild type virus (Figure 5C), a phenotype that has been reported before for ORF73 null virus [[5](#_ENREF_5)].

## Supporting References

## 1. Bochkarev A, Barwell JA, Pfuetzner RA, Furey W, Jr., Edwards AM, et al. (1995) Crystal structure of the DNA-binding domain of the Epstein-Barr virus origin-binding protein EBNA 1. Cell 83: 39-46.

## 2. Hooley E, Fairweather V, Clarke AR, Gaston K, Brady RL (2006) The recognition of local DNA conformation by the human papillomavirus type 6 E2 protein. Nucleic Acids Res 34: 3897-3908.

## 3. Stanley BJ, Ehrlich ES, Short L, Yu Y, Xiao Z, et al. (2008) Structural insight into the human immunodeficiency virus Vif SOCS box and its role in human E3 ubiquitin ligase assembly. J Virol 82: 8656-8663.

## 4. Paden CR, Forrest JC, Tibbetts SA, Speck SH (2012) Unbiased Mutagenesis of MHV68 LANA Reveals a DNA-Binding Domain Required for LANA Function In Vitro and In Vivo. PLoS Pathog 8: e1002906.

## 5. Forrest JC, Paden CR, Allen RD, 3rd, Collins J, Speck SH (2007) ORF73-null murine gammaherpesvirus 68 reveals roles for mLANA and p53 in virus replication. J Virol 81: 11957-11971.
